# A DNA phosphorothioation-based Dnd defense system provides resistance against various phages and is compatible with the Ssp defense system

**DOI:** 10.1128/mbio.00933-23

**Published:** 2023-06-01

**Authors:** Susu Jiang, Ke Chen, Yingying Wang, Yueying Zhang, Yaru Tang, Wanqiu Huang, Xiaolin Xiong, Shi Chen, Chao Chen, Lianrong Wang

**Affiliations:** 1 Department of Gastroenterology, Ministry of Education Key Laboratory of Combinatorial Biosynthesis and Drug Discovery, Hubei Clinical Center and Key Laboratory of Intestinal and Colorectal Disease, Zhongnan Hospital of Wuhan University, School of Pharmaceutical Sciences, Wuhan University, Wuhan, Hubei, China; 2 Department of Respiratory Diseases, The Research and Application Center of Precision Medicine, The Second Affiliated Hospital of Zhengzhou University, Zhengzhou University, Zhengzhou, Henan, China; 3 Department of Burn and Plastic Surgery, Shenzhen Institute of Translational Medicine, Health Science Center, Shenzhen Second People’s Hospital, The First Affiliated Hospital of Shenzhen University, Shenzhen, Guangdong, China; New York University School of Medicine, New York, New York, USA; Michigan State University, East Lansing, Michigan, USA

**Keywords:** DNA phosphorothioation, Dnd systems, Ssp systems, restriction-modification systems, DNA methylation, phage resistance

## Abstract

DndABCDE-catalyzed DNA phosphorothioation (PT), in which the nonbridging oxygen is swapped with a sulfur atom, was first identified in the bacterial genome. Usually, this modification gene cluster is paired with a restriction module consisting of DndF, DndG, and DndH. Although the mechanisms for the antiphage activity conferred by this Dnd-related restriction and modification (R-M) system have been well characterized, several features remain unclear, including the antiphage spectrum and potential interference with DNA methylation. Recently, a novel PT-related R-M system, composed of the modification module SspABCD paired with a single restriction enzyme, SspE, was revealed to be widespread in the bacterial kingdom, which aroused our interest in the interaction between Dnd- and Ssp-based R-M systems. In this study, we discussed the action of Dnd-related R-M systems against phages and demonstrated that the host could benefit from the protection provided by Dnd-related R-M systems against infection by various lytic phages as well as temperate phages. However, this defense barrier would fail against lysogenic phages. Interestingly, DNA methylation, even in the consensus sequence recognized by the Dnd system, could not weaken the restriction efficiency. Finally, we explored the interaction between Dnd- and Ssp-based R-M systems and found that these two systems were compatible. This study not only expands our knowledge of Dnd-associated R-M systems but also reveals a complex interaction between different defense barriers that coexist in the cell.

**IMPORTANCE:**

Recently, we decoded the mechanism of Dnd-related R-M systems against genetic parasites. In the presence of exogenous DNA that lacks PT, the macromolecular machine consisting of DndF, DndG, and DndH undergoes conformational changes to perform DNA binding, translocation, and DNA nicking activities and scavenge the foreign DNA. However, several questions remain unanswered, including questions regarding the antiphage spectrum, potential interference by DNA methylation, and interplay with other PT-dependent R-M systems. Here, we revealed that the host could benefit from Dnd-related R-M systems for a broad range of antiphage activities, regardless of the presence of DNA methylation. Furthermore, we demonstrated that the convergence of Dnd- and Ssp-related R-M systems could confer to the host a stronger antiphage ability through the additive suppression of phage replication. This study not only deepens our understanding of PT-related defense barriers but also expands our knowledge of the arms race between bacteria and their predators.

## INTRODUCTION

Phages, whose population is estimated to be 10^31^ in the biosphere, are responsible for 20%–40% of bacterial mortality every day (1, 2). To respond to such a large number of predators, bacteria have evolved several antiphage strategies targeting different stages of the phage life cycle (3). Innate immune systems, such as DNA methylation-dependent restriction-modification (R-M) systems, bacteriophage exclusion, cyclic-oligonucleotide-based antiphage signaling systems, pyrimidine cyclase systems for anti-phage resistance, and the recently discovered DNA phosphorothioation (PT)-based R-M systems, are triggered by phage components (e.g., phage DNA or proteins) to protect the host or act in an altruistic mode to preserve the population (4 - 12). In contrast to innate immune systems, clustered regularly interspaced short palindromic repeat (CRISPR)–CRISPR-associated protein (Cas) systems, which take advantage of the molecular memory from prior infections, are classified as an adaptive immune mechanism (13). By hijacking the genetic information from the invaders as spacers into the CRISPR array and deploying the repertoire of spacers, the host can complete the acquisition, expression, and interference stages and can thus effectively recognize and eliminate their phage foes.

DNA PT, mediated by DndABCDE, which transfers sulfur atoms from L-cysteine to the microbial genome in a sequence- and stereo-specific manner, was first identified in the bacterial genome, and this system was classified as the Dnd system (14 - 17). To build up a line of defense, the *dndABCDE* gene cluster is usually paired with *dndFGH*, which acts as a restriction module. In the presence of invaders, DndFGH exerts DNA binding, translocation, and nicking activities to initiate the destructive DNA-shredding program to eliminate genetic parasites (18, 19). Interestingly, some DNA methylases, such as Dam, which converts 5′-GATC-3′/5′-GATC-3′ to 5′-G^6m^ATC-3′/5′-G^6m^ATC-3′, can modify the same motif recognized by DndABCDE, leading to the potential conflict with DndFGH in the presence of exogenous DNA harboring a methyl group in the PT modification motif (11, 20 - 22). Recently, a novel PT-associated antiphage system, composed of the modification module SspABCD paired with a single restriction enzyme, SspE, was identified as being widespread in the bacterial kingdom and was classified as an Ssp system. This SspABCD-E system could confer the host with broad-spectrum phage resistance (10, 23 - 25). Unlike the previously characterized Dnd systems, which are responsible for the double-stranded DNA PT modification, Ssp systems confer the host with single-stranded DNA PT modification exclusively at the 5′-C_PS_CA-3′ motifs with much higher frequency. In general, some *dnd* or *ssp* clusters lack *dndA* or *sspA*; DndA or SspA is instead functionally replaced by other cysteine desulfurases, such as IscS, in bacteria.

Although the molecular mechanism underlying the DNA damage caused by DndFGH has been well studied, its phage resistance spectrum has not been characterized. Furthermore, the effect of the convergence of Dnd- and Ssp-related restriction–modification (Dnd R-M, Ssp R-M) systems is less well known. In this study, we determined the spectrum of phage resistance conferred by DndFGH and demonstrated that the Dnd R-M system could protect the host from various lytic phages (T1, T4, T5, T7, and engineered *E. coli* phage EEP) as well as temperate phage λ. Although DndFGH could inhibit phage lysogenization effectively, this defense barrier failed to protect the host on prophage induction. Moreover, by taking advantage of the Dnd R-M systems from *Bermanella marisrubri* RED65, which recognized 5′-GATC-3′/5′-GATC-3′ motifs, we revealed that the methyl group in 5′-G^6m^ATC-3′/5′-G^6m^ATC-3′ would not inhibit the restriction activity of DndFGH. Finally, we combined Dnd R-M and Ssp R-M systems. We found that these two kinds of PT-associated R-M systems were compatible. These findings not only expand our knowledge of PT-associated R-M systems but also deepen our understanding of the arms race between bacteria and phages.

## RESULTS

### The Dnd-related restriction-modification system protects the host from various lytic phages

Recently, we resolved the crystal structure of DndG and the C-terminal domain of DndH (residues 1,313–1,687) from *Escherichia coli* B7A (18). Based on structural and biochemical evidence, we proved that DndF, DndG, and DndH could form a complex with a molar ratio of 2:2:1 and exert DNA binding, translocation, and DNA nicking activities to protect the host from phage invasion (18). However, the antiphage spectrum of DndFGH is unclear.

**Fig 1:**
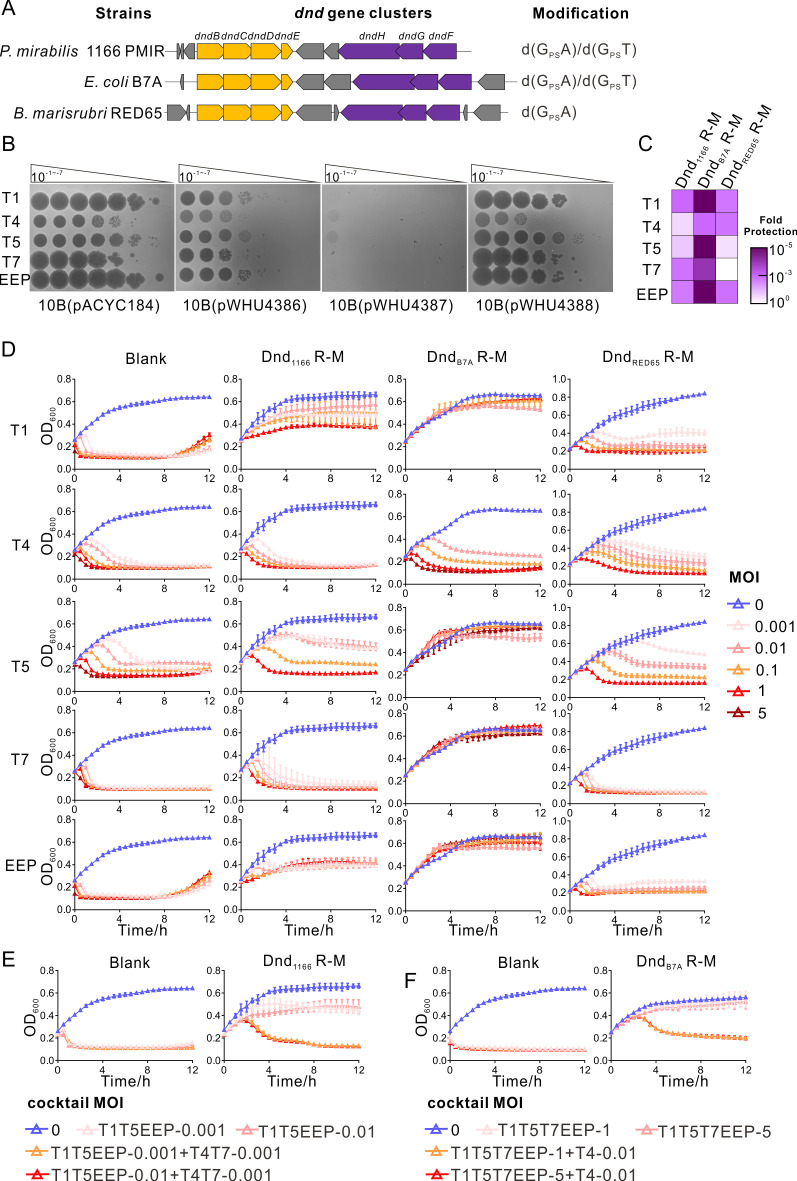
Dnd R-M systems could protect the host from various lytic phages. (**A**) Schematic of *dndBCDE* modules (yellow) paired with *dndFGH* modules (purple) from *P. mirabilis* 1166 PMIR, *E. coli* B7A, and *B. marisrubri* RED65. (**B**) A 5-µL aliquot from a 10-fold serial dilution of phage was spotted onto DH10B harboring pACYC184, pWHU4386, pWHU4387, and pWHU4388. (**C**) Dnd R-M systems exhibited some differences in protection efficiency. The fold protection was assessed by comparing the efficiency of plating (EOP) of phages on DH10B harboring Dnd R-M systems with the EOP on DH10B(pACYC184). (**D**) Growth curves of strains treated with lytic phages at various MOIs. DH10B(pACYC184) was set as the control group, labeled “Blank”. (**E**) and (**F**) Growth curves of strains treated with lytic phage cocktails at various MOIs. The MOI was determined by the individual phages in the phage mix. For example, “T1T5EEP-0.001” represents the phage cocktail composed of T1 (MOI = 0.001), T5 (MOI = 0.001), and EEP (MOI = 0.001), while “T1T5EEP-0.01+T4T7-0.001” represents the phage cocktail composed of T1 (MOI = 0.01), T5 (MOI = 0.01), EEP (MOI = 0.01), T4 (MOI = 0.001), and T7 (MOI = 0.001). All experiments were performed three times.

To determine the antiphage spectrum, we chose three Dnd R-M systems: the Dnd R-M system from *Proteus mirabilis* 1166 PMIR (Dnd_1166_ R-M) with undefined modification motifs, the Dnd R-M system from *E. coli* B7A (Dnd_B7A_ R-M) recognizing 5′-G_PS_AAC-3′/5′-G_PS_TTC-3′, and the Dnd R-M system from *B. marisrubri* RED65 (Dnd_RED65_ R-M) recognizing 5′-G_PS_ATC-3′/5′-G_PS_ATC-3′ (Fig. 1A) (26, 27). First, we cloned *dndBCDE-FGH* derived from *P. mirabilis* 1166 PMIR, *E. coli* B7A, and *B. marisrubri* RED65 into pACYC184, generating pWHU4386, pWHU4387, and pWHU4388, respectively. By applying liquid chromatography with tandem mass spectrometry (LC-MS/MS), d(G_PS_A) and d(G_PS_T) were identified in the genome of *P. mirabilis* 1166 PMIR as well as DH10B(pWHU4386), implying that the motif in Dnd_1166_ was 5′-G_PS_AAC-3′/5′-G_PS_TTC-3′ (Fig. 1A; Fig. S1). As expected, along with the existence of Dnd R-M systems, several protective phenomena, including a decrease in phage titer, a reduction in plaque size, and the absence or delay of bacterial culture collapse, could be observed when the bacteria were challenged with various lytic phages (Fig. 1B and D). To quantify the protection efficiency conferred by Dnd R-M systems, an efficiency of plating (EOP) assay was performed. Consistent with the results from the phage spotting and growth curve assay, Dnd_B7A_ R-M exhibited the strongest phage resistance ability, providing up to five orders of magnitude protection against all the phages tested here. Compared to Dnd_1166_ R-M, which conferred the host with one to three orders of magnitude protection against all the phages tested, Dnd_RED65_ R-M showed the weakest antiphage activity (Fig. 1C; Supplementary Data 1). Interestingly, when coincubated with bacteria in liquid broth, T4 exhibited more aggressive activity against all three Dnd R-M systems than other phages. In addition, Dnd_1166_ R-M was more vulnerable to T7 (Fig. 1D).

The strong phage resistance exhibited by Dnd_1166_ R-M and Dnd_B7A_ R-M prompted us to challenge DH10B(pWHU4386) and DH10B(pWHU4387) with phage cocktails at various multiplicities of infection (MOIs). According to the antiphage performance of Dnd R-M systems during coincubation in the growth curve assay, different phage cocktails were prepared. As shown in Fig. 1E, the Dnd_1166_ R-M systems exhibited activity against the phage cocktails consisting of T1, T5, and EEP at an MOI of 0.01, which has been determined to be the limit of T5 tolerance for Dnd_1166_ R-M systems. However, with the addition of T4 and T7, even at an MOI of 0.001, which is the lowest concentration that we tested, the growth curve collapsed similarly to that of the control group. Again, Dnd_B7A_ R-M systems exhibited strong antiphage activity against the phage cocktails consisting of T1, T5, T7, and EEP even at an MOI of 5, which was the highest concentration that we tested. However, consistent with the phenomenon that we observed in Dnd_1166_ R-M systems, the growth curve collapsed when strains carrying Dnd_B7A_ R-M systems were challenged with the all-in-one phage cocktail (Fig. 1F).

These data demonstrated that the Dnd R-M systems could protect their host from various lytic phages, even when facing allied phage forces.

### The Dnd system could protect the host from lysogenization of λ but not prophage induction

Compared to the lytic phage, temperate phages, such as λ, which can remain dormant as prophages in *E. coli* and become reactivated to achieve proliferation and lyse the host, have a more complex life cycle. This prompted us to investigate the potential resistance ability conferred by the Dnd R-M system against λ during the process of lysogenization as well as prophage induction.

**Fig 2:**
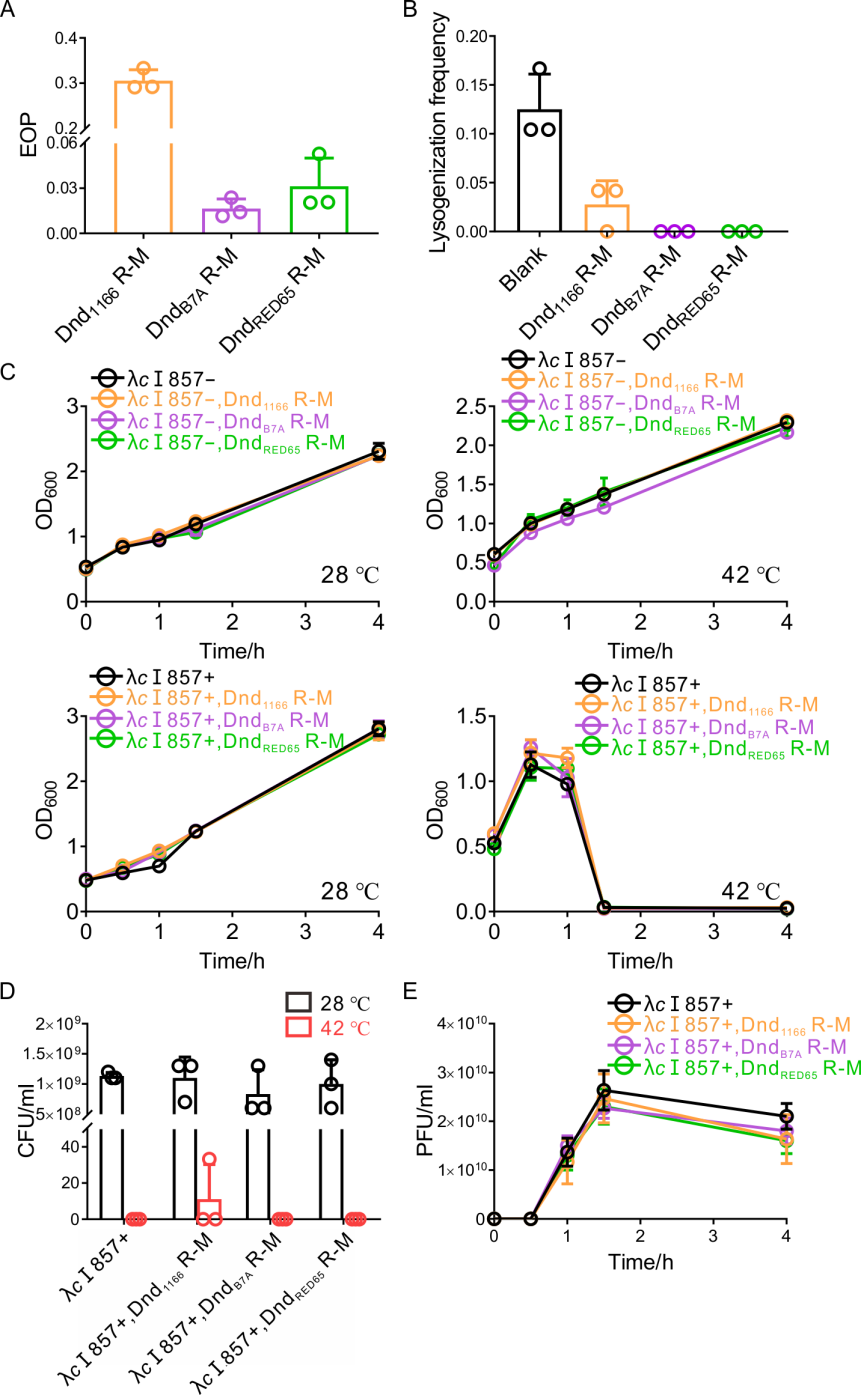
Dnd R-M protects the host from the lysogenization of λ but not prophage induction. (**A**) The EOP of λ on strains equipped with different Dnd R-M systems. (**B**) The Dnd R-M system could protect the host from the lysogenization of λ. (**C**) Growth curves of strains harboring different Dnd R-M systems without the lysogenization of λcI857 incubated at 28°C or 42°C (upper). Growth curves of lysogens parasitized with λcI857 and carrying different Dnd R-M systems incubated at 28°C or 42°C (lower). (**D**) The number of survivors for the lysogens incubated at 28°C or 42°C. (**E**) Variation in the number of viral progeny released from lysogens, which carried different Dnd R-M systems, after the temperature shift from 28°C to 42°C. All experiments were performed three times.

First, we evaluated the EOP for phage λ invading the strains harboring Dnd_B7A_, Dnd_1166_, or Dnd_RED65_ R-M systems. Phage λ could generate visible plaques on DH10B(Dnd_B7A_ R-M), DH10B(Dnd_1166_ R-M), and DH10B(Dnd_RED65_ R-M) with EOPs of (1.64 ± 0.58) × 10^−2^, (3.05 ± 0.22) × 10^−1^, and (3.13 ± 1.67) × 10^−2^, respectively, indicating that the Dnd R-M system could protect the host from invasion by temperate phages such as λ (Fig. 2A). After identifying the λ resistance ability conferred by Dnd R-M, we began to evaluate the effectiveness of Dnd R-M in preventing phage lysogenization. To generate lysogens, we mixed DH10B with phage λ at an MOI of 1 and incubated it on LB agar plates. Colonies were checked by PCR to identify successful lysogenization. The efficiency of lysogenization reached 12.50% ± 3.23% for DH10B without the protective effect of Dnd R-M. For DH10B(Dnd_1166_ R-M), the lysogenization efficiency was reduced to 2.78% ± 2.15%, and no lysogens were detected in strains harboring Dnd_B7A_ or Dnd_RED65_ R-M (Fig. 2B). These observations indicated that Dnd R-M systems could prevent the temperate phage from lysogenizing the host.

The other characteristic that distinguished temperate phages from lytic phages is that the prophage persisting as dormant components of host cells could be “woken up” to lyse the host cells. For some defense systems, such as CRISPR-Cas systems, the phage DNA can be eliminated during prophage induction. Thus, the parasitized hosts can be “cured” (28). However, no such data are available for Dnd R-M systems. Therefore, we examined the potential effect of Dnd R-M on this “internal foe”. For efficient induction, we used λ*c*I857 instead of λ. This phage can be triggered at 42°C due to the temperature-sensitive *c*I mutation (29 - 31). Unlike the CRISPR-Cas defense barrier, Dnd R-M systems failed to provide effective protection when we changed the temperature from 28°C to 42°C. As shown in Fig. 2C, the presence of λ*c*I857 at 28°C or the Dnd system at 42°C did not influence the growth rate of DH10B. In sharp contrast, the OD_600_ dropped steeply to nearly 0 after 1.5 hours at the elevated temperature and remained steady in all the strains harboring λ*c*I857 regardless of the existence of Dnd R-M systems. Furthermore, we calculated the survival rate by plating the bacteria on LB agar plates and incubating them at 28°C or 42°C. After induction, the survival rates were (0 ± 0), (1.01 ± 1.56) × 10^−8^, (0 ± 0), and (0 ± 0) for strains carrying pACYC184, pWHU4386, pWHU4387, and pWHU4388, respectively (Fig. 2D). Meanwhile, we monitored progeny production by λ*c*I857 during induction. For strains equipped with Dnd R-M systems, λ*c*I857 began to lyse the cells at 30 minutes and released ~25 viral particles per cell during the first 1.5 hours after temperature shift, which was consistent with the phenomenon we observed in DH10B(λ*c*I857, pACYC184) (Fig. 2E).

Considering that the efficiency of the Dnd R-M system could be impaired due to the rise in temperature (11), the λ phage resistance ability of Dnd R-M at 28°C and 42°C was assessed to rule out the potential influence of temperature variation. We found that the rise in temperature did not impair the protection efficiency of the Dnd_1166_ or Dnd_B7A_ R-M systems, and the EOP was (3.05 ± 0.22) × 10^−1^ or (3.26 ± 0.63) × 10^−1^ and (1.64 ± 0.58) × 10^−2^ or (1.93 ± 0.71) × 10^−2^ for the Dnd_1166_ and Dnd_B7A_ R-M systems at 28°C or 42°C, respectively. However, the EOP increased by approximately one order of magnitude from (3.13 ± 1.67) × 10^−2^ to (3.64 ± 0.47) × 10^−1^ for the Dnd_RED65_ R-M systems as the temperature increased. Considering that this value was close to that from Dnd_1166_ R-M, we hypothesized that this weakened defense system could still provide sufficient protection (Fig. 2A; Fig. S2). Thus, the lack of protection against prophage induction was not caused by the temperature variation.

In this section, we provided evidence that Dnd R-M systems could protect the host from lysogenization of λ but not prophage induction.

### DNA methylation does not interfere with the phage resistance provided by DndFGH

Previously, we demonstrated *in vivo* that DndABCDE might share a consensus sequence with the coexisting DNA methylase*,* generating a hybrid modification structure (11, 27). This overlap among different R-M systems introduces the possibility that DNA methylation might impair the function of DndFGH, suggesting that exogenous DNA carrying a methyl group at the appropriate position might be protected from DndFGH. To test this hypothesis, we chose Dnd_RED65_ R-M, which recognizes 5′-G_PS_ATC-3′/5′-G_PS_ATC-3′, and Dam methyltransferase, generating 5′-G^6m^ATC-3′/5′-G^6m^ATC-3′ to build the model.

First, we evaluated the transformation efficiency of strains equipped with Dnd_RED65_ R-M using pBluescript II SK+ as substrates. Plasmids were prepared from *E. coli* BW25113 and the associated *dam*-deficient strain JW3350 and verified by using MboI and DpnI, respectively (32) (Fig. S3A). Unlike the Dnd_1166_ and Dnd_B7A_ R-M systems, no restriction effect has been observed for Dnd_RED65_ R-M systems. The transformation efficiency of strains harboring Dnd_RED65_ R-M systems against pBluescript II SK+ carrying 5′-GATC-3′/5′-GATC-3′ or 5′-G^6m^ATC-3′/5′-G^6m^ATC-3′ was (4.08 ± 0.15) × 10^−4^ or (4.68 ± 0.29) × 10^−4^, respectively, which was similar to the situation in DH10B(pACYC184) ([4.30 ± 0.30] × 10^−4^ versus [6.09 ± 1.52] × 10^−4^) (Fig. S3B).

**Fig 3:**
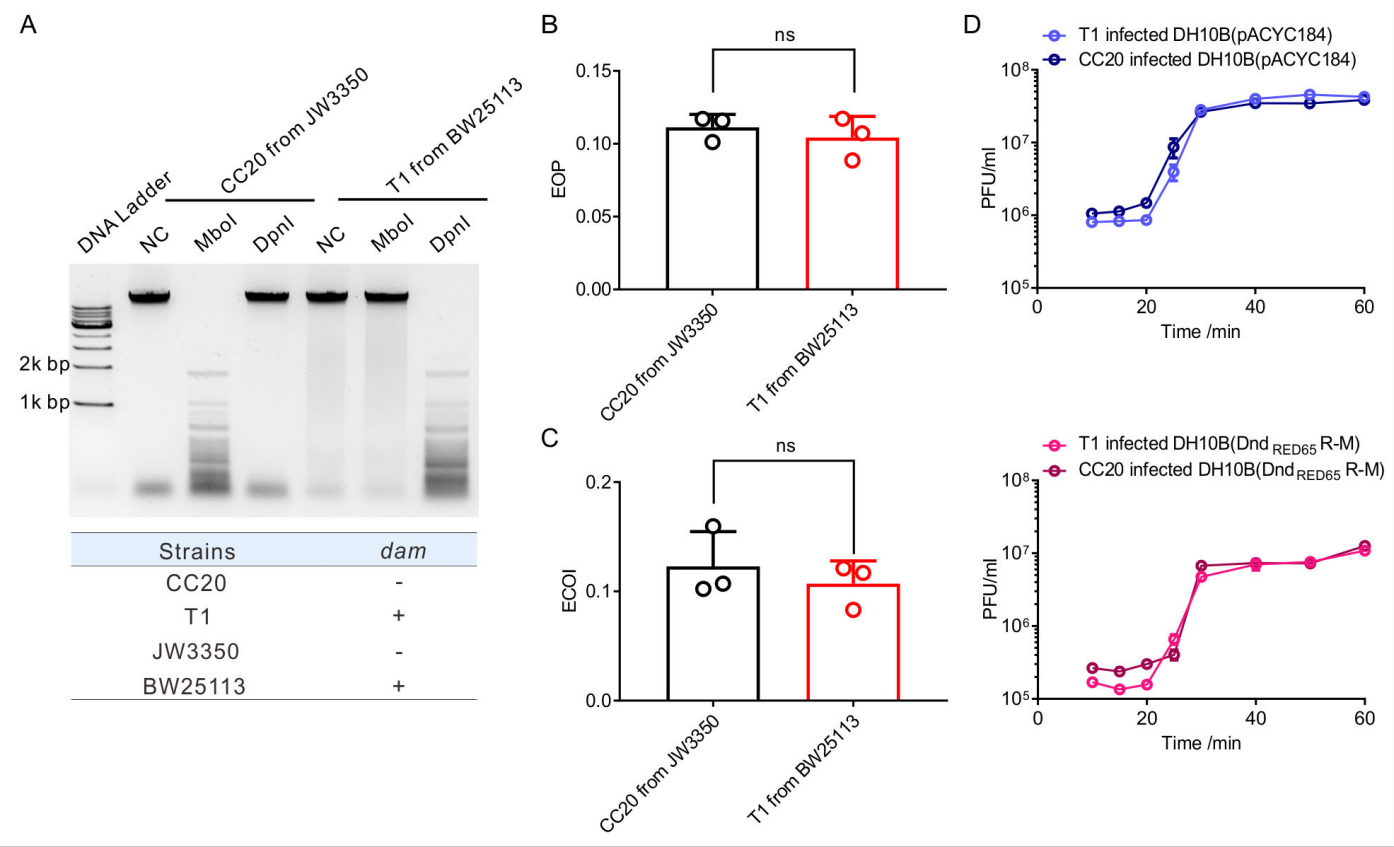
DNA methylation could not impair the restriction of Dnd R-M systems. (**A**) The digestion of DNA isolated from T1 and CC20 proliferated in BW25113 or JW3350 with MboI or DpnI. All the 5′-GATC-3′/5′-GATC-3′ motifs on CC20, which is a *dam*-deficient strain derived from T1 and prepared from JW3350, remained unmethylated. (**B**) The EOP of T1 and CC20, prepared from BW25113 and JW3350, respectively, on DH10B(Dnd_RED65_ R-M). (**C**) The efficiency of the center of infection (ECOI) of T1 and CC20, prepared from BW25113 and JW3350, respectively, on DH10B(Dnd_RED65_ R-M). (**D**) One-step growth curves of T1 and CC20, prepared from BW25113 and JW3350, respectively, when infected with DH10B(pACYC184) (upper) or DH10B(Dnd_RED65_ R-M) (lower). All the experiments were performed three times. NC, negative control; ns, not significant.

This result led us to use T1 phages to evaluate the response of Dnd_RED65_ R-M systems to methylated DNA. First, we determined the modification motif of the DNA methylase (protein_ID = YP_003925.1) encoded by the T1 phage. The genomic DNA of T1 prepared from JW3350 remained sensitive to DpnI, indicating that this Dam methyltransferase could convert 5′-GATC-3′/5′-GATC-3′ to 5′-G^6m^ATC-3′/5′-G^6m^ATC-3′, which is consistent with previous research (33, 34) (Fig. S4C). To prepare T1 with a “naked” genome, we deleted *dam* from the T1 genome using the CRISPR-Cas system to generate CC20 (Fig. S4A and B). The enzyme digestion assay demonstrated that the genomic DNA from CC20 prepared from JW3350 lost resistance to DpnI and showed restored sensitivity to MboI, indicating that all the 5′-GATC-3′ sites in the CC20 genome remained unmethylated (Fig. 3A). On challenging DH10B(pACYC184) or DH10B(Dnd_RED65_ R-M) with T1 or CC20, the EOP as well as the efficiency of the center of infection (ECOI) were determined by calculating the ratio of the phage titer measured in DH10B(Dnd_RED65_ R-M) to that measured in DH10B(pACYC184) (Fig. 3B and C). Surprisingly, DH10B(Dnd_RED65_ R-M) showed similar resistance to both CC20 and T1, with an EOP of (1.11 ± 0.07) × 10^−1^ versus (1.04 ± 0.13) × 10^−1^ and an ECOI of (1.23 ± 0.28) × 10^−1^ versus (1.07 ± 0.19) × 10^−1^, respectively, implying that DndFGH could scavenge invaders regardless of DNA methylation. Furthermore, to explore the potential influence of the Dnd_RED65_ R-M system on phage proliferation in a single life cycle in more detail, one-step growth curves were generated for DH10B(pACYC184) and DH10B(Dnd_RED65_ R-M) infected with CC20 and T1, respectively (Fig. 3D). Compared with that of T1, the burst size of CC20 decreased to approximately 60% (48.75 ± 8.65 versus 29.24 ± 3.02) on infection of DH10B(Dnd_RED65_ R-M). However, without the protection of the Dnd_RED65_ R-M system, the burst sizes of T1 and CC20 were 52.26 ± 3.10 and 31.93 ± 2.73, respectively, indicating that the decrease was caused by *dam* deletion rather than the effect of the Dnd_RED65_ R-M system. These data demonstrated that DNA methylation would not interfere with the action of Dnd R-M systems even if both systems recognized the consensus sequence. Surprisingly, the presence of Dnd_RED65_ R-M systems showed no significant influence on the burst size of T1 phage (*P* value = 0.9235), which seemed contradictory to the previous results (18). We proposed that this phenomenon could be attributed to the relatively weak defense activity of Dnd_RED65_ R-M. To verify this hypothesis, we rechecked the one-step growth curve of T1 when DH10B(Dnd_B7A_ R-M), which exhibited the strongest protection, was treated as the host. Consistent with the previous results, Dnd_B7A_ R-M conferred the host with powerful protection with an ECOI of (3.12 ± 0.27) × 10^−3^, indicating that there was only a narrow chance for T1 to produce at least one active progeny in the presence of Dnd_B7A_ R-M. However, the burst size of T1 decreased slightly from 52.26 ± 3.10 to 40.65 ± 5.95 (*P* value 0.0185) (Fig. S5A and B). These data indicated that the Dnd R-M system exhibited antiphage activity mainly in the early stage of phage infection. Once the escaped phages launched the replication process, the protection conferred by DndFGH was limited.

### The Dnd restriction–modification system could be compatible with SspABCD-E

Recently, we discovered a novel Ssp R-M system that consists of SspABCD-E (10). The single restriction enzyme SspE could sense the sulfur atom on 5′-C_PS_CA-3′ generated by SspABCD and exert nicking activity toward unmodified DNA, thus protecting the host from various phages (23). The different mechanisms of the Dnd- and Ssp-based defense barriers prompted us to examine the compatibility of these two kinds of systems.

**Fig 4:**
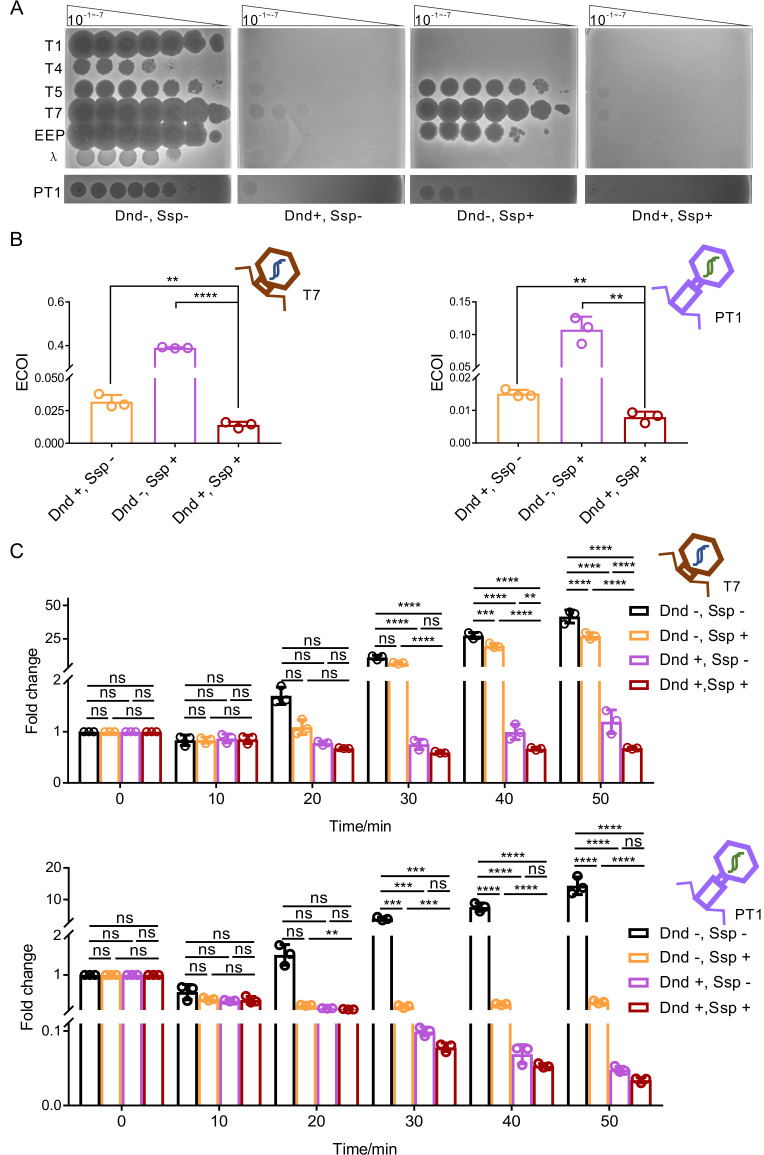
Dnd R-M systems could be compatible with Ssp R-M systems. (**A**) A 5-µL aliquot of a 10-fold serial dilution of phage was spotted onto *E. coli* or *S. enterica* strains harboring Dnd and/or Ssp R-M systems. (**B**) ECOI of T7 or PT1 on *E. coli* or *S. enterica* strains harboring Dnd and/or Ssp R-M systems, respectively. (**C**) Time course for the DNA replication of T7 or PT1 in *E. coli* or *S. enterica* strains harboring Dnd and/or Ssp R-M systems, respectively. All experiments were performed three times. **P < 0.01, ***P < 0.001, ****P < 0.0001, ns, not significant.

To address this subject, we chose *E. coli* MG1655-PT, which was derived by integrating *sspBCD-E* from *E. coli* 3234/A into the genome of MG1655, as well as *Salmonella enterica* serovar Cerro 87, which carried *dndBCDE-FGH*, as model strains. We introduced pACYC184, pWHU4386, pWHU4387, and pWHU4388 into MG1655 or MG1655-PT. Additionally, we transferred pACYC184 and pWHU3638, which were derived from pACYC184 and carried *sspBCD-E* from *E. coli* 3234/A into *S. enterica* serovar Cerro 87 as well as the associated *dndB-H*-deficient strain XTG103 to generate strains with four different statuses: (Dnd−, Ssp−), (Dnd+, Ssp−), (Dnd−, Ssp+), and (Dnd+, Ssp+). First, we determined the growth rate of these strains to check for potential conflict between these two kinds of PT-based systems. Similar growth curves were observed using either LB broth or M9 broth, suggesting that the combination of these two kinds of PT-related R-M systems did not inhibit cell growth (Fig. S6). Consistent with the previous results, the Dnd R-M systems exhibited strong phage resistance, and phage plaques could hardly be seen on LB plates coated with Dnd R-M system-armed cells (Fig. 4A). An additive phenomenon could be observed for MG1655 harboring both Dnd and Ssp R-M systems (Fig. 4A; Fig. S7A and B). Since the additive effect could hardly be seen in the *S. enterica* strains, which might have been due to the overpowering phage resistance conferred by the endogenous Dnd R-M system, we calculated the ECOI to assess the effect of this combination in *S. enterica*. Compared to the protection from Dnd R-M or Ssp R-M alone against the PT1 phage, which is a lytic *S. enterica* phage, with ECOIs of (1.51 ± 0.11) × 10^−2^ and (1.07 ± 0.18) × 10^−1^, respectively, the combination of these two systems significantly decreased the ECOI to (7.94 ± 1.48) × 10^−3^ (Fig. 4B). Similar phenomena were observed in the MG1655-series strains when we challenged the strains with T7 phages (Fig. 4B).

The increase in phage resistance caused by the combination of the Dnd and Ssp R-M systems prompted us to perform an qRT-PCR assay, which is an effective and simple method (9, 35), to evaluate the phage DNA replication rates in the strains harboring different defense systems. In sharp contrast to the rapid replication of phage DNA in the strain lacking the protection of PT-related R-M systems, the presence of Dnd R-M or Ssp R-M alone significantly reduced or inhibited phage DNA replication. Further enhancement in protection efficiency could be observed with the convergence of these two kinds of defense barriers (Fig. 4C). Although there was no statistically significant difference between Cerro 87(pACYC184) and Cerro 87(pWHU3638), the DNA replication rate of PT1 from Cerro 87(pWHU3638) was consistently slower than that of PT1 from Cerro 87(pACYC184) from 30 minutes after infection, implying an additive defense effect due to the coexistence of Dnd and Ssp R-M systems.

## DISCUSSION

In this study, we proved that similar to Ssp R-M systems, DndABCDE-FGH can provide a solid defense against various phages, even phage cocktails. Interestingly, the antiphage and antiplasmid preferences were quite different among different Dnd R-M systems, even when they shared the same DNA motifs. However, although DndFGH exhibited strong resistance to invasion by temperate phages, such as λ, this fortress was not infallible against attack by “internal enemies”. This was reasonable considering the action mode of R-M systems, which distinguish self- and nonself-DNA based on the specific modification tags. A prophage that is successfully integrated into the genome is recognized as self-DNA and modified as well as replicated together with the lysogen. With the protection provided by the PT modification, prophages can escape restriction by Dnd R-M systems. Notably, researchers recently proved that the PT modification could influence the transcription of the genes encoded by the prophage and alter the binding affinity of the repressor associated with prophage activation, indicating a more complex scenario of the interaction between Dnd systems and prophages (36).

Surprisingly, the methyl group modified in the consensus motifs recognized by Dnd systems did not impair the restriction efficiency of DndFGH, although there was a conflict between Dam methylase and DndABCDE (27). This phenomenon supported the idea that DndFGH exclusively sensed PT tags. However, another possibility could not be ignored: although the full methylation of the T1 genome could be proved by enzyme digestion, few unmethylated 5′-GATC-3′/5′-GATC-3′ motifs, which were randomly distributed in the T1 genome, could be detected using [^3^H]-labeled AdoMet (34). These unmodified motifs might become potential targets for DndFGH. Interestingly, the deletion of *dam* from the T1 genome was responsible for the decrease in phage burst sizes even in the strains containing Dam of bacterial origin (Protein ID: NP_417846.1). Although these two Dam methylases could both modify 5′-GATC-3′/5′-GATC-3′ to generate 5′-G^6m^ATC-3′/5′-G^6m^ATC-3′, no significant sequence similarity was found between these two kinds of Dam methylases. This prompted us to check the modification status of the CC20 progeny released from BW25113 (Fig. S4C). Based on the findings that (i) the DNA was still sensitive to MboI, although higher bands could be observed compared to those from the phages prepared from JW3350 and (ii) the DNA was also sensitive to DpnI but remained more intact than the DNA from the MboI-treated group, we proposed that the T1 phage DNA could only be partially modified by the Dam of bacterial origin, which has been proven to be a highly efficient methyltransferase (37). The methylation status might be associated with the efficiency of progeny production.

In addition, we demonstrated that Dnd and Ssp R-M could work together to suppress phage DNA replication. It is worth mentioning that it is possible to apply this combination strategy in more species due to the widespread distribution of Dnd and Ssp systems in the bacterial kingdom, including in *Streptomyces*, *Bacillus*, and *Halomonas*, which can be used as microbial chassis for industrial products. Recently, we identified a novel defense module consisting of SspFGH, which shared no significant sequence identity with DndFGH, and the combination of SspFGH with SspE could confer the host with stronger protection against phages (38). These compatibility phenomena revealed the high inclusivity of the PT-based R-M systems and the potential for developing these basic elements as an antiphage package in the field of biosynthesis.

**Fig 5:**
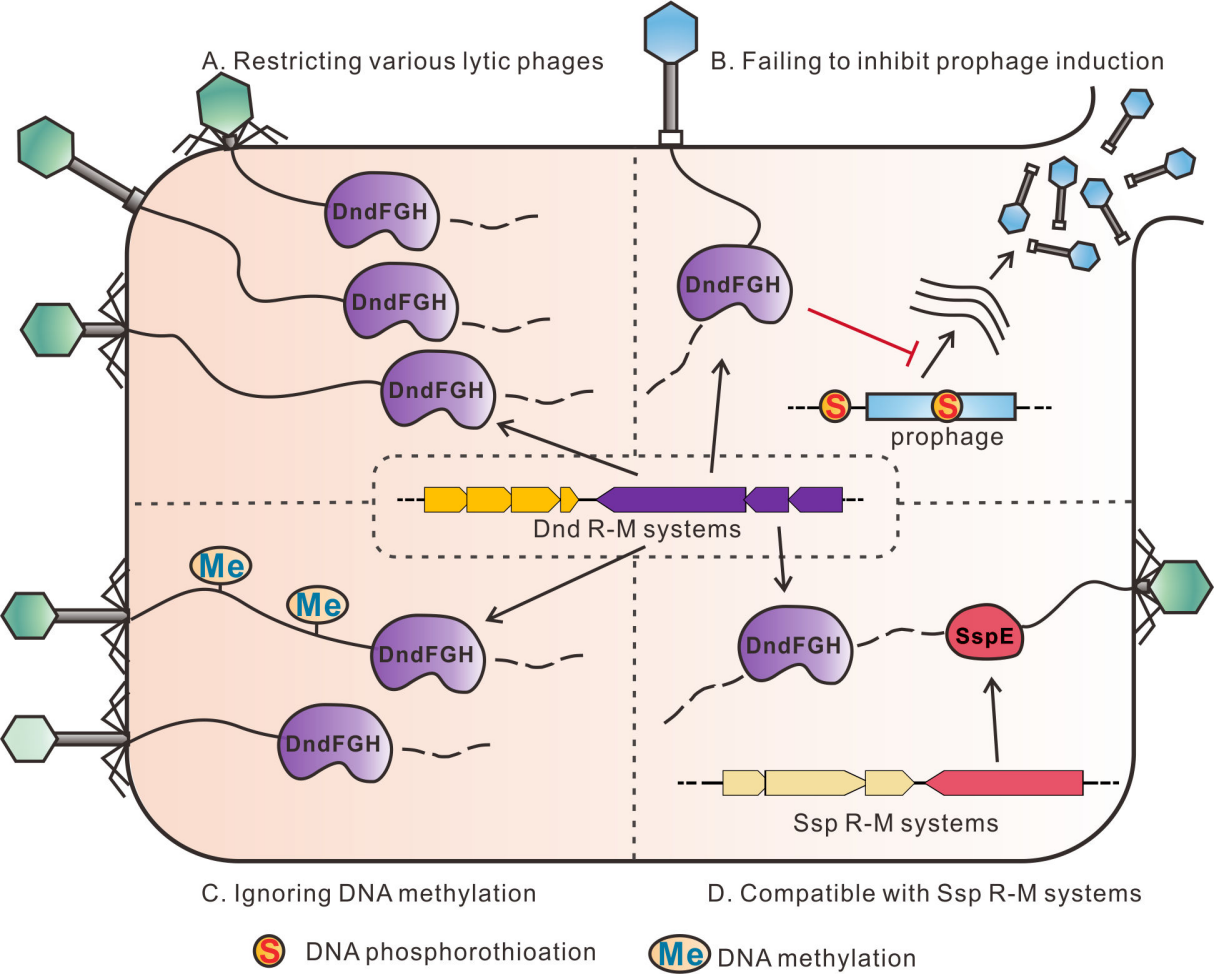
A schematic model of the Dnd R-M system against phage infection. Dnd R-M systems protect the host from various lytic phages as well as the lysogenization of temperate phages (**A**), but not prophage induction (**B**). (**C**) The methylation of phage genomic DNA does not interfere with phage resistance by DndFGH. (**D**) The convergence of Dnd and Ssp R-M systems could confer the host with stronger antiphage ability.

Taken together, the results demonstrated the strong antiphage activities provided by Dnd R-M systems, covering a broad spectrum of phages, and showed that DndFGH could overcome steric clashes due to DNA methylation. In addition, PT-related R-M systems could coexist and be compatible with each other (Fig. 5). These data not only deepen our understanding of PT-based R-M systems but also describe the complex interplay between bacteria and their phage predators.

## MATERIALS AND METHODS

### Bacterial strains, bacteriophages, plasmids, and media

All of the strains, bacteriophages, and plasmids used in this study are listed in Table S1 and Table S2. The primers used in this study are listed in Table S2. The *Escherichia coli* and *Salmonella enterica* strains were routinely grown at 37°C or 28°C in Luria-Bertani (LB) broth or on LB agar plates unless otherwise indicated. *Proteus mirabilis* strains were grown in LB broth at 37°C. *B. marisrubri* strains were grown in DSMZ Medium 514c at 28°C. When necessary, an appropriate concentration of antibiotic (100 µg/mL ampicillin and/or 25 µg/mL chloramphenicol) was added to the medium.

### Plasmid construction

For the construction of pWHU4386, a 16,029-bp fragment containing the *dndBCDEFGH* gene cluster was amplified from the genomic DNA of *P. mirabilis* 1166 PMIR using the primer pair 1166F/1166R (Table S2). The fragment was inserted into the HindIII-BamHI-digested pACYC184 plasmid using a ClonExpress Ultra One Step Cloning Kit (Vazyme), generating pWHU4386.

For the construction of pWHU4387, the 8,286-bp and 8,321-bp fragments containing the whole *dndBCDEFGH* gene cluster were amplified from the genomic DNA of *E. coli* B7A using the primer pairs B7A-LL/B7A-LR and B7A-RL/B7A-RR (Table S2), respectively. The two fragments were inserted into the HindIII–BamHI-digested pACYC184 plasmid through Gibson assembly cloning, yielding pWHU4387.

For the construction of pWHU4388, the 8,137-bp and 7,637-bp fragments containing the whole *dndBCDEFGH* gene cluster were amplified from the genomic DNA of *B. marisrubri* RED65 using the primer pairs RED-LL/RED-LR and RED-RL/RED-RR (Table S2), respectively. The two fragments were inserted into the HindIII–BamHI-digested pACYC184 plasmid through Gibson assembly cloning.

For the construction of pWHU3638, an 8,069-bp fragment containing the *sspBCDE* gene cluster was amplified from the genomic DNA of *E. coli* 3234/A using the primer pair 3234F/3234R (Table S2). The fragment was inserted into the SalI-BamHI-digested pACYC184 plasmid, generating pWHU3638.

For the construction of pWHU710, both 66-bp homologous sequences were amplified from the genomic DNA of T1 phage using the primer pairs Dam-LL/Dam-LR and Dam-RL/Dam-RR (Table S2). The two fragments were inserted into the HindIII-BamHI-digested pBluescript II SK+ through Gibson assembly cloning, yielding pWHU710.

For the construction of pWHU711, first, the spacer targeting *dam* of T1 was prepared by annealing the primer pair Spacer-F/Spacer-R (Table S2). Then, the spacer was ligated into BsaI-digested pCas9 using T4 DNA ligase (NEB), generating pWHU711.

### Construction of CC20

Overnight-cultured *E. coli* DH10B(pWHU710, pWHU711) was diluted 1:100 in LB medium containing 100 µg/mL ampicillin and 25 µg/mL chloramphenicol at 37°C until the optical density at 600 nm (OD_600_) reached 0.6–0.8. A 300-µL bacterial culture was mixed with molten soft agar (0.75%) and immediately overlaid on prepoured LB agar (1.5%) plates. Phage T1 stocks were subjected to 10-fold serial dilution with SM buffer (100 mM NaCl, 8 mM MgSO_4_, and 50 mM Tris-Cl pH 7.5) and spotted on the upper layer of the plate. After incubation overnight at 37°C, plaques were verified by PCR using the primer pair Dam-LL/Dam-RR (Table S2). The mutants were purified twice and further confirmed by DNA sequencing.

### Phage plaque assays

Overnight-cultured *E. coli* or *S. enterica* strains were diluted 1:100 in LB medium containing the appropriate antibiotic at 37°C or 28°C until the OD_600_ reached 0.6–0.8. A 300-µL bacterial culture was mixed with molten soft agar (0.75%) and immediately overlaid on prepoured LB agar (1.5%) plates. Phage stocks were subjected to 10-fold serial dilution with SM buffer. Once the top agar solidified, 5 µL of each 10-fold diluted phage stock was spotted onto the bacterial lawns and cultured overnight at the indicated temperatures.

### Efficiency of plating (EOP) assay

Overnight-cultured *E. coli* strains were diluted 1:100 in LB medium containing the appropriate antibiotic and incubated until they reached the log phase (~OD_600_ = 0.6–0.8). The molten soft agar (0.75%) containing 100 µL of serially diluted phage solution and 300 µL of mid-exponential bacterial culture was poured onto a preprepared LB agar (1.5%) plate and incubated overnight at a fixed temperature. EOP was calculated by dividing PFU/mL (on the tested strain) by PFU/mL (on the control strain).

### Efficiency of the center of infection (ECOI) assay

Overnight-cultured *E. coli* or *S. enterica* strains were diluted 1:100 in LB broth containing 1 mM CaCl_2_, 1 mM MgCl_2_, and the appropriate antibiotic at 37°C or 28°C until the OD_600_ reached 0.5. One milliliter of the bacterial culture was mixed with phage solution to meet the multiplicity of infection (MOI) of 0.1. The mixture was incubated without shaking for 5 minutes for phage absorption. After centrifugation at 10,000× *g* for 1 minute, the pellet was washed twice with LB broth and resuspended in 1 mL of LB. Samples were subjected to 10-fold serial dilution, mixed with molten soft agar (0.75%) containing the phage-sensitive host, poured onto preprepared LB agar (1.5%) plates, and incubated overnight. The ECOI was calculated by dividing the number of COIs on the sensitive strain by the number of COIs on the resistant strain.

### One-step growth curve assay

Overnight-cultured *E. coli* strains were diluted 1:100 in LB broth containing the appropriate antibiotic, 1 mM CaCl_2_, and 1 mM MgCl_2_ and incubated at 37°C until the OD_600_ reached 0.6. Phage was added to the log-phase cells to achieve a final MOI of 0.01. After a 5-minute incubation, 1 mL of cell cultures was centrifuged at 10,000× *g* for 1 minute to remove the unabsorbed phages. The pellet was washed twice, resuspended in 1 mL of LB, subjected to 10-fold serial dilution, and incubated at 37°C. Then, 100 µL aliquots were taken periodically over 60 minutes from the appropriate dilution gradient, mixed with molten soft agar (0.75%) containing DH10B and overlaid onto preprepared LB agar (1.5%) plates. The burst size of the phage was determined by dividing the PFU/mL in the latent period by the PFU/mL in the plateau period.

### Bacterial growth curve assay

Overnight-cultured *E. coli* or *S. enterica* strains were diluted 1:100 in LB or M9 medium containing the appropriate antibiotic and incubated until the OD_600_ reached 0.5. The log-phase cultures were diluted 1:100 in LB or M9 medium and transferred into a 96-well plate to monitor the growth of samples using a microplate spectrophotometer (Synergy H1MF, BioTek). The OD_600_ of each sample was measured at 30-minute intervals during the incubation. For the phage infection growth curve, phages were added at various MOIs to log-phase (OD_600_ = 0.5) cells and cocultured in a 96-well plate to monitor the growth of the cells by a microplate spectrophotometer (Synergy H1MF, BioTek).

### Transformation efficiency assay

Fifty nanograms of pBluescript II SK+ isolated from *E. coli* BW25113 or JW3350 was transformed in parallel into chemically competent cells with or without *dndBCDE-FGH* modules. After overnight cultivation on LB agar plates with or without ampicillin, the transformation efficiency was measured by dividing CFU/mL (from plates with ampicillin) by CFU/mL (from plates without ampicillin).

### Quantification of real-time PCR assay

Log-phase (OD_600_ = 0.5) *E. coli* or *S. enterica* strains were infected with phage T7 at an MOI of 0.5 or phage PT1 at an MOI of 5. Samples were harvested at each time point, and DNA was extracted using the chloroform–phenol method. For each quantitative RT-PCR (qRT-PCR), 1 ng of total DNA was added as a template. The primer pairs RT-T7-F/RT-T7-R and RT-E-gapA-F/RT-E-gapA-R were used to monitor the abundance of DNA from T7 and *E. coli*, respectively. For the quantification of DNA abundance from *S. enterica* and PT1, the primer pairs RT-PT1-F/RT-PT1-R and RT-S-gapA-F/RT-S-gapA-R were used instead (Table S2). Real-time PCR was performed using Taq Pro Universal SYBR qPCR Master Mix (Vazyme) and a QuantStudio 3 Real-Time PCR System (ThermoFisher Scientific) to determine the threshold cycle (Ct). The comparative Ct (2^−ΔΔCt^) method was applied to determine the relative DNA level.

### LC-MS/MS analysis of PT modifications

*P. mirabilis* and *E. coli* strains were incubated until the OD_600_ reached 0.6–0.8. Genomic DNA was extracted, hydrolyzed, dephosphorylated, and detected by liquid chromatography with tandem mass spectrometry (LC-MS/MS) analysis, as previously described (26). Briefly, 20 μg of bacterial genomic DNA, which was extracted using the chloroform-phenol method, was digested with nuclease P1 followed by dephosphorylation with alkaline phosphatase. After ultrafiltration with a Nanoseq 10K column (PALL, USA), the filtrate was loaded onto a Thermo Hypersil Gold aQ column (150 × 2.1 mm, 3 µm) coupled to a Thermo TSQ Quantum Access MAX mass spectrometer for PT modification detection. Elution was performed at a flow rate of 0.2 mL/minute with solvent A (water containing 0.1% acetic acid) and solvent B (acetonitrile with 0.1% acetic acid) according to the following profile: 97% solvent A for 5 minutes, followed by 97%–94% solvent A over 42 minutes and 94%–2% solvent A in 1 minute.

### Lambda lysogenization assay

λ and DH10B were mixed at an MOI of 1 and incubated at room temperature for 15 minutes. The unabsorbed phages were removed by centrifugation at 10,000× *g* for 1 minute. The culture was diluted to an appropriate gradient and overlaid on LB agar (1.5%) plates. After incubation for 24 hours at 28°C, the colonies were checked by PCR using the primer pair Lambda-F/Lambda-R (Table S2) to detect successful lysogenization.

### Lambda induction assay

λcI857 instead of λ was applied in this assay. The overnight-cultured lysogen carrying λcI857 was diluted 1:100 in LB with appropriate antibiotics and incubated at 28°C until the OD_600_ reached 0.5.

To assess the survival rate of bacteria under the induction effect of λ*c*I857, cultures were subjected to 10-fold serial dilution and parallelly overlaid on LB agar (1.5%) plates. The plates were incubated at 28°C or 42°C. The survival rate was calculated by dividing CFU/mL (from plates incubated at 42°C) by CFU/mL (from plates incubated at 28°C).

To evaluate the growth rate of the bacteria and the progeny production efficiency, 1 mL of culture was taken at each time point to monitor the OD_600_ (ND-100C, MIULAB) after the temperature was shifted to 42°C. Meanwhile, bacteria were removed by centrifugation at 10,000× *g* for 1 minute. The supernatant was subjected to 10-fold serial dilution, mixed with DH10B in molten soft agar (0.75%), and immediately overlaid on prepoured LB agar (1.5%) plates. The PFU/mL was calculated after overnight incubation.

## SUPPLEMENTAL MATERIAL

FIG. S1 mbio.00933-23-s0001.docx DndBCDE from *P. mirabilis* 1166 PMIR is responsible for d(G_PS_A)/d(G_PS_T) in *R*_P_.

FIG. S2 mbio.00933-23-s0002.docx Dnd R-M systems could confer protection to the host against λ at 42°C.

FIG. S3 mbio.00933-23-s0003.docx Transformation efficiency of SK+ into strains with different Dnd R-M systems.

FIG. S4 mbio.00933-23-s0004.docx The Dam of bacterial origin could only partially modify the genome of CC20.

FIG. S5 mbio.00933-23-s0005.docx Restriction efficiency of Dnd_B7A_ R-M against T1.

FIG. S6 mbio.00933-23-s0006.docx The growth curves of *E. coli* or *S. enterica* strains harboring Dnd and/or Ssp R-M systems in LB or M9 medium.

FIG. S7 mbio.00933-23-s0007.docx Combination of Dnd_1166_ R-M or Dnd_RED65_ R-M with SspBCD-E.

TABLE S1 mbio.00933-23-s0008.docx Strains and phages used in this study.

TABLE S2 mbio.00933-23-s0009.docx Plasmids and primers used in this study.

Data S1 mbio.00933-23-s0010.xls Dnd protection against phages in *E. coli* DH10B.

ASM does not own the copyrights to Supplemental Material that may be linked to, or accessed through, an article. The authors have granted ASM a non-exclusive, world-wide license to publish the Supplemental Material files. Please contact the corresponding author directly for reuse.

## References

[1] Fortier LC , Sekulovic O . 2013. Importance of prophages to evolution and virulence of bacterial pathogens. Virulence 4:354–365. doi:10.4161/viru.24498 23611873 PMC3714127

[2] Suttle CA . 2007. Marine viruses -- major players in the global ecosystem. Nat Rev Microbiol 5:801–812. doi:10.1038/nrmicro1750 17853907

[3] Hampton HG , Watson BNJ , Fineran PC . 2020. The arms race between bacteria and their phage foes. Nature 577:327–336. doi:10.1038/s41586-019-1894-8 31942051

[4] Loenen WAM , Dryden DTF , Raleigh EA , Wilson GG , Murray NE . 2014. Highlights of the DNA cutters: a short history of the restriction enzymes. Nucleic Acids Res 42:3–19. doi:10.1093/nar/gkt990 24141096 PMC3874209

[5] Goldfarb T , Sberro H , Weinstock E , Cohen O , Doron S , Charpak-Amikam Y , Afik S , Ofir G , Sorek R . 2015. BREX is a novel phage resistance system widespread in microbial genomes. EMBO J 34:169–183. doi:10.15252/embj.201489455 25452498 PMC4337064

[6] Cohen D , Melamed S , Millman A , Shulman G , Oppenheimer-Shaanan Y , Kacen A , Doron S , Amitai G , Sorek R . 2019. Cyclic GMP-AMP signalling protects bacteria against viral infection. Nature 574:691–695. doi:10.1038/s41586-019-1605-5 31533127

[7] Huiting E , Cao X , Ren J , Athukoralage JS , Luo Z , Silas S , An N , Carion H , Zhou Y , Fraser JS , Feng Y , Bondy-Denomy J . 2023. Bacteriophages inhibit and evade cGAS-like immune function in bacteria. Cell 186:864–876. doi:10.1016/j.cell.2022.12.041 36750095 PMC9975087

[8] Tal N , Morehouse BR , Millman A , Stokar-Avihail A , Avraham C , Fedorenko T , Yirmiya E , Herbst E , Brandis A , Mehlman T , Oppenheimer-Shaanan Y , Keszei AFA , Shao S , Amitai G , Kranzusch PJ , Sorek R . 2021. Cyclic CMP and cyclic UMP mediate bacterial immunity against phages. Cell 184:5728–5739. doi:10.1016/j.cell.2021.09.031 34644530 PMC9070634

[9] Xiong L , Liu S , Chen S , Xiao Y , Zhu B , Gao Y , Zhang Y , Chen B , Luo J , Deng Z , Chen X , Wang L , Chen S . 2019. A new type of DNA phosphorothioation-based antiviral system in archaea. Nat Commun 10: 1688. doi:10.1038/s41467-019-09390-9 30975999 PMC6459918

[10] Xiong X , Wu G , Wei Y , Liu L , Zhang Y , Su R , Jiang X , Li M , Gao H , Tian X , Zhang Y , Hu L , Chen S , Tang Y , Jiang S , Huang R , Li Z , Wang Y , Deng Z , Wang J , Dedon PC , Chen S , Wang LR . 2020. SspABCD-SspE is a phosphorothioation-sensing bacterial defence system with broad anti-phage activities. Nat Microbiol 5:917–928. doi:10.1038/s41564-020-0700-6 32251370

[11] Chen C , Wang L , Chen S , Wu X , Gu M , Chen X , Jiang S , Wang Y , Deng Z , Dedon PC , Chen S . 2017. Convergence of DNA methylation and phosphorothioation epigenetics in bacterial genomes. Proc Natl Acad Sci U S A 114:4501–4506. doi:10.1073/pnas.1702450114 28400512 PMC5410841

[12] Xu T , Yao F , Zhou X , Deng Z , You D . 2010. A novel host-specific restriction system associated with DNA backbone S-modification in Salmonella. Nucleic Acids Res 38:7133–7141. doi:10.1093/nar/gkq610 20627870 PMC2978375

[13] Barrangou R , Fremaux C , Deveau H , Richards M , Boyaval P , Moineau S , Romero DA , Horvath P . 2007. CRISPR provides acquired resistance against viruses in prokaryotes. Science 315:1709–1712. doi:10.1126/science.1138140 17379808

[14] Wang L , Chen S , Xu T , Taghizadeh K , Wishnok JS , Zhou X , You D , Deng Z , Dedon PC . 2007. Phosphorothioation of DNA in bacteria by dnd genes. Nat Chem Biol 3:709–710. doi:10.1038/nchembio.2007.39 17934475

[15] Cao B , Chen C , DeMott MS , Cheng Q , Clark TA , Xiong X , Zheng X , Butty V , Levine SS , Yuan G , Boitano M , Luong K , Song Y , Zhou X , Deng Z , Turner SW , Korlach J , You D , Wang L , Chen S , Dedon PC . 2014. Genomic mapping of phosphorothioates reveals partial modification of short consensus sequences. Nat Commun 5: 3951. doi:10.1038/ncomms4951 24899568 PMC4322921

[16] Wang LR , Jiang SS , Deng ZX , Dedon PC , Chen S . 2019. DNA phosphorothioate modification-a new multi-functional epigenetic system in bacteria. FEMS Microbiol Rev 43:109–122. doi:10.1093/femsre/fuy036 30289455 PMC6435447

[17] Xia SS , Chen J , Liu LQ , Wei Y , Deng ZX , Wang LR , Chen S . 2019. Tight control of genomic phosphorothioate modification by the ATP-modulated autoregulation and reusability of DndB. Mol Microbiol 111:938–950. doi:10.1111/mmi.14186 30552823

[18] Wu D , Tang Y , Chen S , He Y , Chang X , Zheng W , Deng Z , Li Z , Wang L , Wu G , Chen S . 2022. The functional coupling between restriction and DNA phosphorothioate modification systems underlying the DndFGH restriction complex. Nat Catal 5:1131–1144. doi:10.1038/s41929-022-00884-2

[19] Gan R , Wu X , He W , Liu Z , Wu S , Chen C , Chen S , Xiang Q , Deng Z , Liang D , Chen S , Wang L . 2014. Dna phosphorothioate modifications influence the global transcriptional response and protect DNA from double-stranded breaks. Sci Rep 4: 6642. doi:10.1038/srep06642 25319634 PMC4198939

[20] Marinus MG , Casadesus J . 2009. Roles of DNA adenine methylation in host-pathogen interactions: mismatch repair, transcriptional regulation, and more. FEMS Microbiol Rev 33:488–503. doi:10.1111/j.1574-6976.2008.00159.x 19175412 PMC2941194

[21] Wion D , Casadesús J . 2006. N6-methyl-adenine: an epigenetic signal for DNA–protein interactions. Nat Rev Microbiol 4:183–192. doi:10.1038/nrmicro1350 16489347 PMC2755769

[22] Adhikari S , Curtis PD . 2016. DNA methyltransferases and epigenetic regulation in bacteria. FEMS Microbiol Rev 40:575–591. doi:10.1093/femsre/fuw023 27476077

[23] Gao H , Gong X , Zhou J , Zhang Y , Duan J , Wei Y , Chen L , Deng Z , Wang J , Chen S , Wu G , Wang L . 2022. Nicking mechanism underlying the DNA phosphorothioate-sensing antiphage defense by SspE. Nat Commun 13:6773. doi:10.1038/s41467-022-34505-0 36351933 PMC9646914

[24] Zou X , Xiao X , Mo Z , Ge Y , Jiang X , Huang R , Li M , Deng Z , Chen S , Wang L , Lee SY . 2022. Systematic strategies for developing phage resistant Escherichia coli strains. Nat Commun 13:4491. doi:10.1038/s41467-022-31934-9 35918338 PMC9345386

[25] Liu L , Jiang S , Xing M , Chen C , Lai C , Li N , Liu G , Wu D , Gao H , Hong L , Tan P , Chen S , Deng Z , Wu G , Wang L , Chapman MR . 2020. Structural analysis of an L-cysteine desulfurase from an Ssp DNA phosphorothioation system. mBio 11: e00488-20. doi:10.1128/mBio.00488-20 32345643 PMC7188994

[26] Wang L , Chen S , Vergin KL , Giovannoni SJ , Chan SW , DeMott MS , Taghizadeh K , Cordero OX , Cutler M , Timberlake S , Alm EJ , Polz MF , Pinhassi J , Deng Z , Dedon PC . 2011. DNA phosphorothioation is widespread and quantized in bacterial genomes. Proc Natl Acad Sci U S A 108:2963–2968. doi:10.1073/pnas.1017261108 21285367 PMC3041111

[27] Wu X , Cao B , Aquino P , Chiu T-P , Chen C , Jiang S , Deng Z , Chen S , Rohs R , Wang L , Galagan JE , Dedon PC . 2020. Epigenetic competition reveals density-dependent regulation and target site plasticity of phosphorothioate epigenetics in bacteria. Proc Natl Acad Sci U S A 117:14322–14330. doi:10.1073/pnas.2002933117 32518115 PMC7322087

[28] Edgar R , Qimron U . 2010. The Escherichia coli CRISPR system protects from λ lysogenization, lysogens, and prophage induction. J Bacteriol 192:6291–6294. doi:10.1128/JB.00644-10 20889749 PMC2981215

[29] Valdez-Cruz NA , Caspeta L , Pérez NO , Ramírez OT , Trujillo-Roldán MA . 2010. Production of recombinant proteins in E. coli by the heat inducible expression system based on the phage lambda pL and/or pR promoters. Microb Cell Fact 9: 18. doi:10.1186/1475-2859-9-18 20298615 PMC2848208

[30] Fu L , Lu C . 2013. A novel dual vector coexpressing PhiX174 lysis E gene and staphylococcal nuclease A gene on the basis of lambda promoter pR and pL, respectively. Mol Biotechnol 54:436–444. doi:10.1007/s12033-012-9581-0 22782703

[31] Villaverde A , Benito A , Viaplana E , Cubarsi R . 1993. Fine regulation of ci857-controlled gene expression in continuous culture of recombinant Escherichia coli by temperature. Appl Environ Microbiol 59:3485–3487. doi:10.1128/aem.59.10.3485-3487.1993 8250569 PMC182479

[32] Baba T , Ara T , Hasegawa M , Takai Y , Okumura Y , Baba M , Datsenko KA , Tomita M , Wanner BL , Mori H . 2006. Construction of Escherichia coli K-12 in-frame, single-gene knockout mutants: the Keio collection. Mol Syst Biol 2:2006.0008. doi:10.1038/msb4100050 PMC1681482 16738554

[33] Schneider-Scherzer E , Auer B , de Groot EJ , Schweiger M . 1990. Primary structure of a DNA (N6-Adenine) -methyltransferase from Escherichia coli virus T1. DNA sequence, genomic organization, and comparative analysis. J Biol Chem 265:6086–6091. doi:10.1016/S0021-9258(19)39295-6 2180941

[34] Scherzer E , Auer B , Schweiger M . 1987. Identification, purification, and characterization of Escherichia coli virus T1 DNA Methyltransferase. J Biol Chem 262:15225–15231. doi:10.1016/S0021-9258(18)48162-8 3312202

[35] Bari SMN , Chou-Zheng L , Howell O , Hossain M , Hill CM , Boyle TA , Cater K , Dandu VS , Thomas A , Aslan B , Hatoum-Aslan A . 2022. A unique mode of nucleic acid immunity performed by a multifunctional bacterial enzyme. Cell Host Microbe 30:570–582. doi:10.1016/j.chom.2022.03.001 35421352

[36] Jian H , Xu G , Yi Y , Hao Y , Wang Y , Xiong L , Wang S , Liu S , Meng C , Wang J , Zhang Y , Chen C , Feng X , Luo H , Zhang H , Zhang X , Wang L , Wang Z , Deng Z , Xiao X . 2021. The origin and impeded dissemination of the DNA phosphorothioation system in prokaryotes. Nat Commun 12: 6382. doi:10.1038/s41467-021-26636-7 34737280 PMC8569181

[37] Barras F , Marinus MG . 1989. The great GATC: DNA methylation in E. coli. Trends Genet 5:139–143. doi:10.1016/0168-9525(89)90054-1 2667217

[38] Wang S , Wan M , Huang R , Zhang Y , Xie Y , Wei Y , Ahmad M , Wu D , Hong Y , Deng Z , Chen S , Li Z , Wang L , Keim P . 2021. SspABCD-sspFGH constitutes a new type of DNA phosphorothioate-based bacterial defense system. mBio 12: e00613-21. doi:10.1128/mBio.00613-21 33906925 PMC8092258

