# Supplementary material for: A DNA phosphorothioation-based Dnd defense system provides resistance against various phages and is compatible with the Ssp defense system
Source: mBio. 2023 Jun 1;14(4):e00933-23. doi: 10.1128/mbio.00933-23 (PMC10470545; doi:10.1128/mbio.00933-23)

**Fig. S1 DndBCDE from *P. mirabilis* 1166 PMIR is responsible for d(G_PS_A)/d(G_PS_T) in *R*_P_.** Synthesized d(X_PS_Y) (X or Y represent A, T, C or G) in the *R*_P_ configuration was used as a standard to identify the modification motif of the Dnd system from *P. mirabilis* 1166 PMIR. pWHU4386 was generated by integrating *dndBCDE-FGH* from *P. mirabilis* 1166 PMIR into pACYC184. By applying HPLC‒MS/MS, d(G_PS_A) as well as d(G_PS_T) in *R*_P_ could be detected from the DNA isolated from *P. mirabilis* 1166 PMIR and DH10B(pWHU4386). RA, relative abundance.


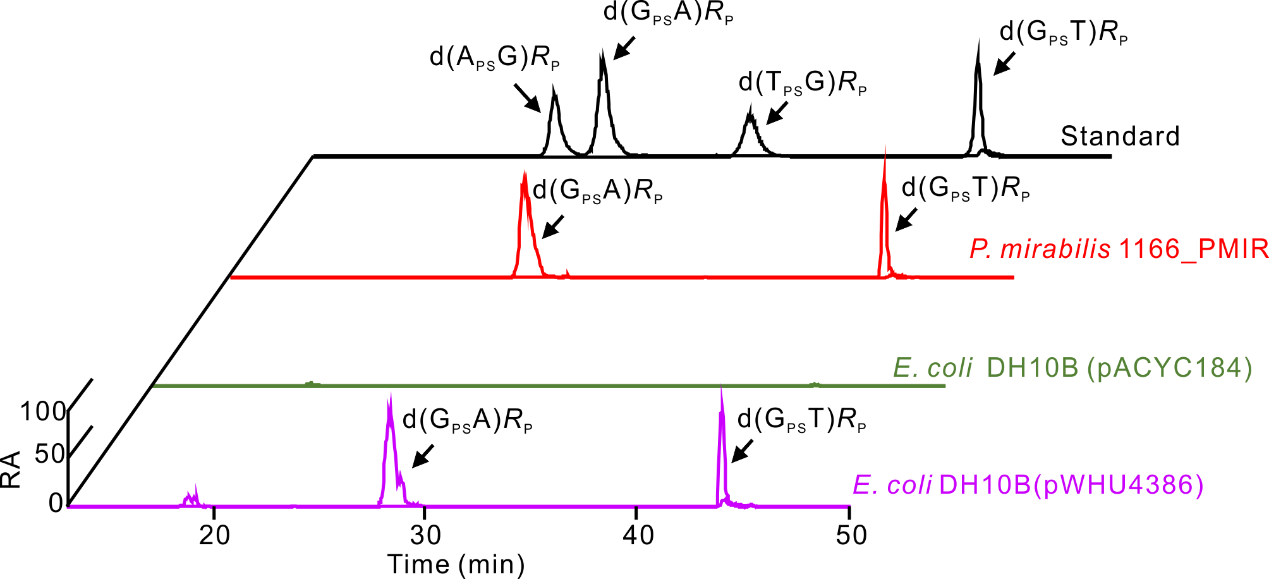

Supplement: FIG. S1 — DndBCDE from P. mirabilis 1166 PMIR is responsible for d(GPSA)/d(GPST) in RP. [file mbio.00933-23-s0001.docx]
