# Supplementary material for: A DNA phosphorothioation-based Dnd defense system provides resistance against various phages and is compatible with the Ssp defense system
Source: mBio. 2023 Jun 1;14(4):e00933-23. doi: 10.1128/mbio.00933-23 (PMC10470545; doi:10.1128/mbio.00933-23)

**Fig. S2 Dnd R-M systems could confer protection to the host against λ at 42**°C**.** The EOP of λ on DH10B carrying Dnd_1166_ R-M, Dnd_B7A_ R-M or Dnd_RED65_ R-M at 42°C. All experiments were performed three times.


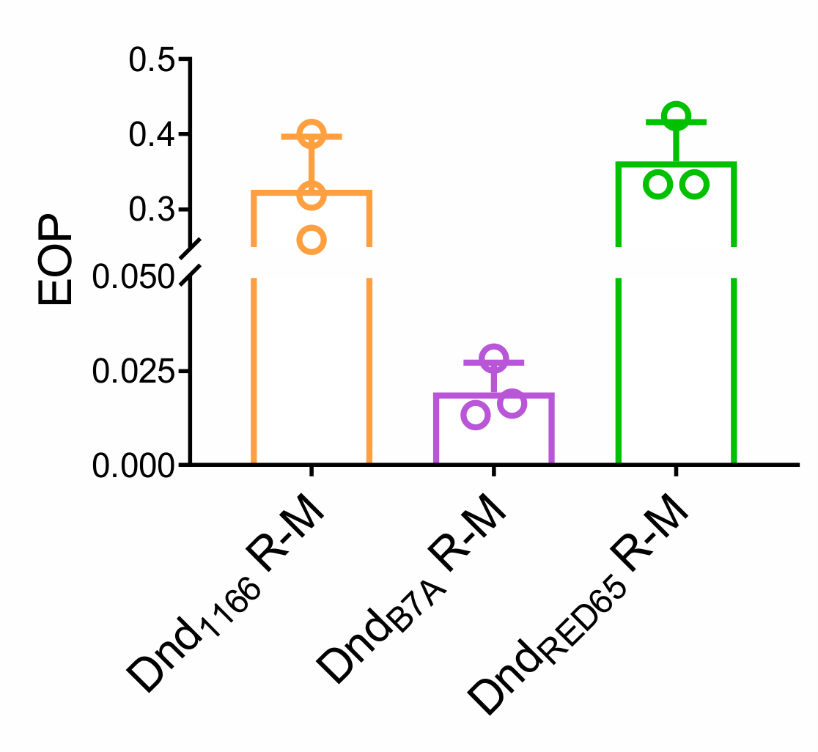

Supplement: FIG. S2 — Dnd R-M systems could confer protection to the host against λ at 42°C. [file mbio.00933-23-s0002.docx]
