# Supplementary material for: A DNA phosphorothioation-based Dnd defense system provides resistance against various phages and is compatible with the Ssp defense system
Source: mBio. 2023 Jun 1;14(4):e00933-23. doi: 10.1128/mbio.00933-23 (PMC10470545; doi:10.1128/mbio.00933-23)

**Fig. S3 Transformation efficiency of SK+ into strains with different Dnd R-M systems. (A)** DNA digestion of SK+ prepared from BW25113 or JW3350 with MboI or DpnI. The digestion pattern of SK+ isolated from JW3350 indicates that all the 5’-GATC-3’/5’-GATC-3’ motifs remained unmethylated. **(B)** The transformation efficiency of SK+ prepared from JW3350 (red) or BW25113 (black) into strains with different Dnd R-M systems. All the experiments were performed four times. NC, negative control.


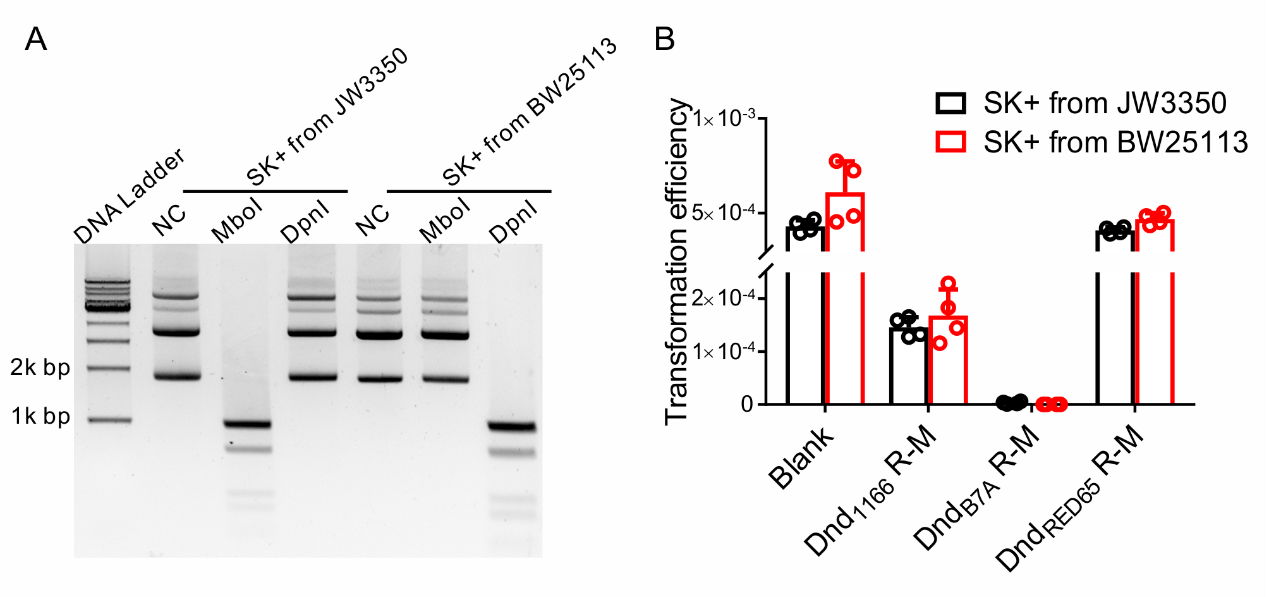

Supplement: FIG. S3 — Transformation efficiency of SK+ into strains with different Dnd R-M systems. [file mbio.00933-23-s0003.docx]
