# Supplementary material for: A DNA phosphorothioation-based Dnd defense system provides resistance against various phages and is compatible with the Ssp defense system
Source: mBio. 2023 Jun 1;14(4):e00933-23. doi: 10.1128/mbio.00933-23 (PMC10470545; doi:10.1128/mbio.00933-23)
Supplement: FIG. S4 — The Dam of bacterial origin could only partially modify the genome of CC20. [file mbio.00933-23-s0004.docx]

**Fig. S4 The Dam of bacterial origin could only partially modify the genome of CC20. (A)** Gene context of *dam* on the genome of T1. The white block represents the deletion region that we designed. The blue arrows represent the primers used to verify the *dam* deletion. **(B)** PCR verified the *dam* deletion from the T1 genome. **(C)** DNA digestion of T1 and CC20, prepared from BW25113 or JW3350, with MboI or DpnI. NC, negative control.

**
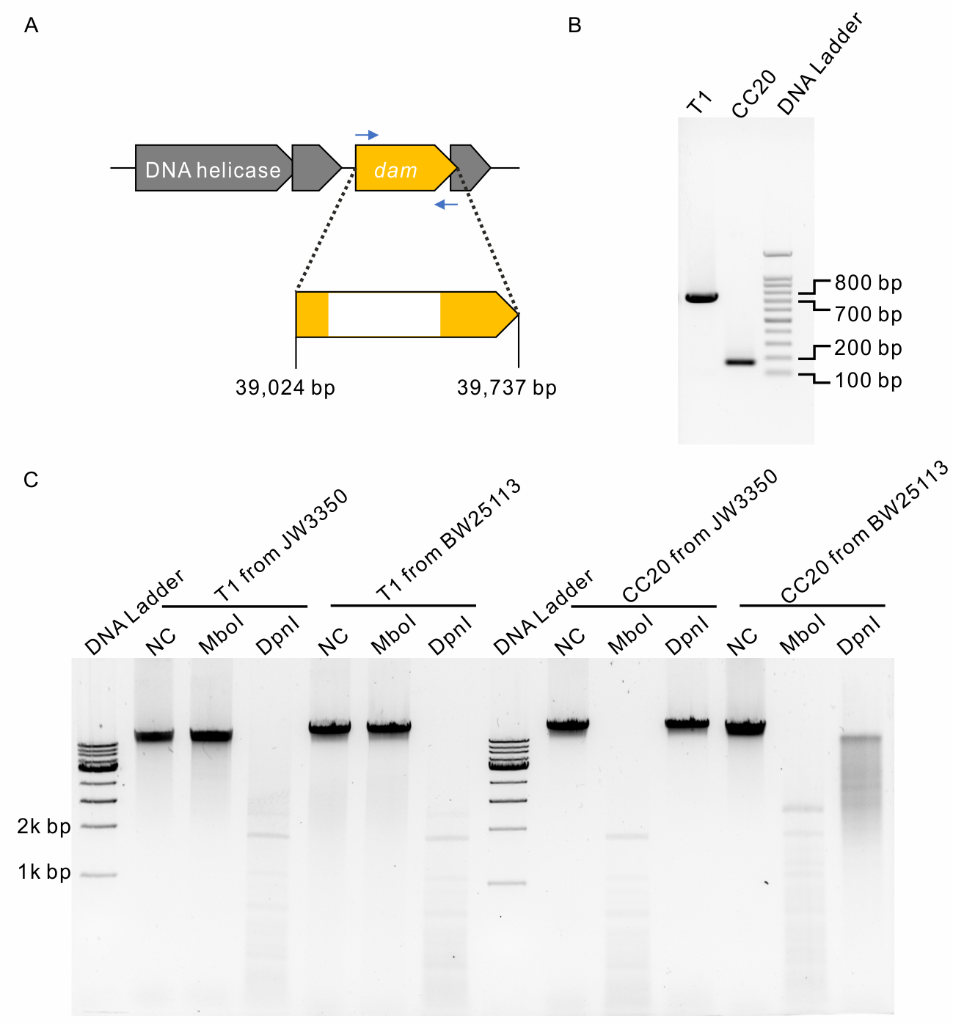
**
