# Supplementary material for: A DNA phosphorothioation-based Dnd defense system provides resistance against various phages and is compatible with the Ssp defense system
Source: mBio. 2023 Jun 1;14(4):e00933-23. doi: 10.1128/mbio.00933-23 (PMC10470545; doi:10.1128/mbio.00933-23)

**Fig. S5 Restriction efficiency of Dnd_B7A_ R-M against T1. (A)** One-step growth curves of T1 infecting DH10B(pACYC184) or DH10B(Dnd_B7A_ R-M). **(B)** The burst size of T1 in DH10B(pACYC184) or DH10B(Dnd_B7A_ R-M). All the experiments were performed four times. *, *P*<0.05.


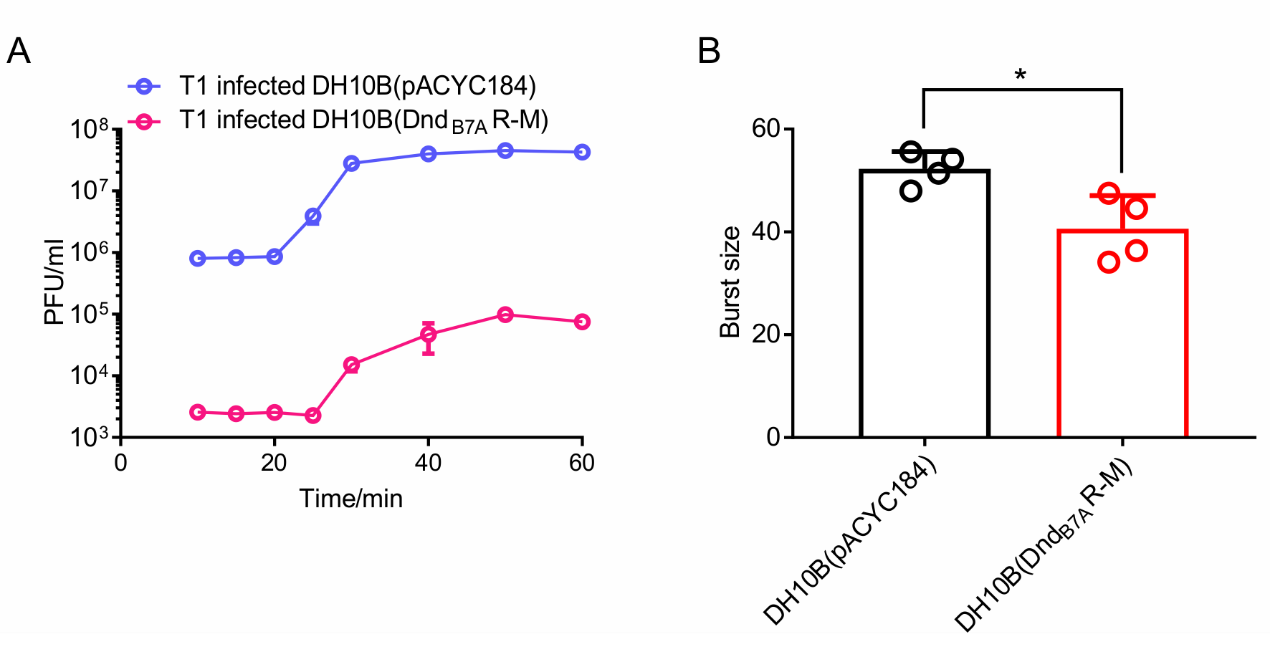

Supplement: FIG. S5 — Restriction efficiency of DndB7A R-M against T1. [file mbio.00933-23-s0005.docx]
