# Supplementary material for: A DNA phosphorothioation-based Dnd defense system provides resistance against various phages and is compatible with the Ssp defense system
Source: mBio. 2023 Jun 1;14(4):e00933-23. doi: 10.1128/mbio.00933-23 (PMC10470545; doi:10.1128/mbio.00933-23)

**Fig. S6 The growth curves of *E. coli* or *S. enterica* strains harboring Dnd and/or Ssp R-M systems in LB or M9 medium.** The combination of Ssp with Dnd_Sal_ R-M **(A)**, Dnd_1166_ R-M **(B)**, Dnd_B7A_R-M **(C)** or Dnd_RED65_ R-M **(D)** did not influence the cell growth in LB or M9 medium. Dnd_Sal_ R-M represents the *dndBCDE-FGH* from *S. enterica*.


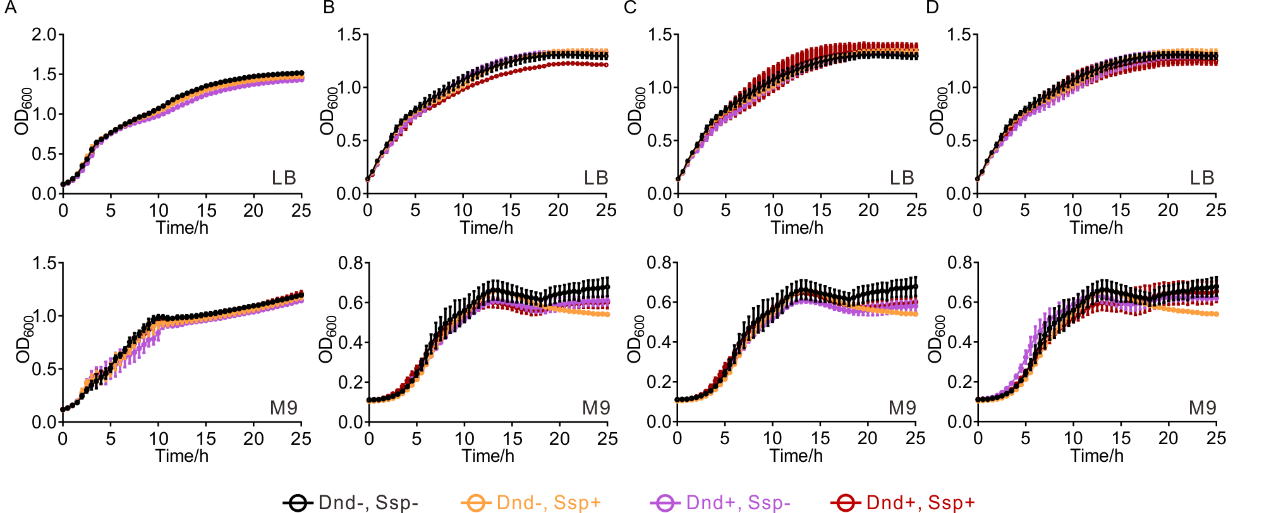

Supplement: FIG. S6 — The growth curves of E. coli or S. enterica strains harboring Dnd and/or Ssp R-M systems in LB or M9 medium. [file mbio.00933-23-s0006.docx]
