# Supplementary material for: A DNA phosphorothioation-based Dnd defense system provides resistance against various phages and is compatible with the Ssp defense system
Source: mBio. 2023 Jun 1;14(4):e00933-23. doi: 10.1128/mbio.00933-23 (PMC10470545; doi:10.1128/mbio.00933-23)
Supplement: FIG. S7 — Combination of Dnd1166 R-M or DndRED65 R-M with SspBCD-E. [file mbio.00933-23-s0007.docx]

**Fig. S7 Combination of Dnd_1166_ R-M or Dnd_RED65_ R-M with SspBCD-E.** A 5 μl of aliquot of a tenfold serial dilution of phage was spotted onto *E. coli* combining Dnd_1166_ R-M **(A)** or Dnd_RED65_ R-M **(B)** with *sspBCD-E* from *E. coli* 3234/A.

**
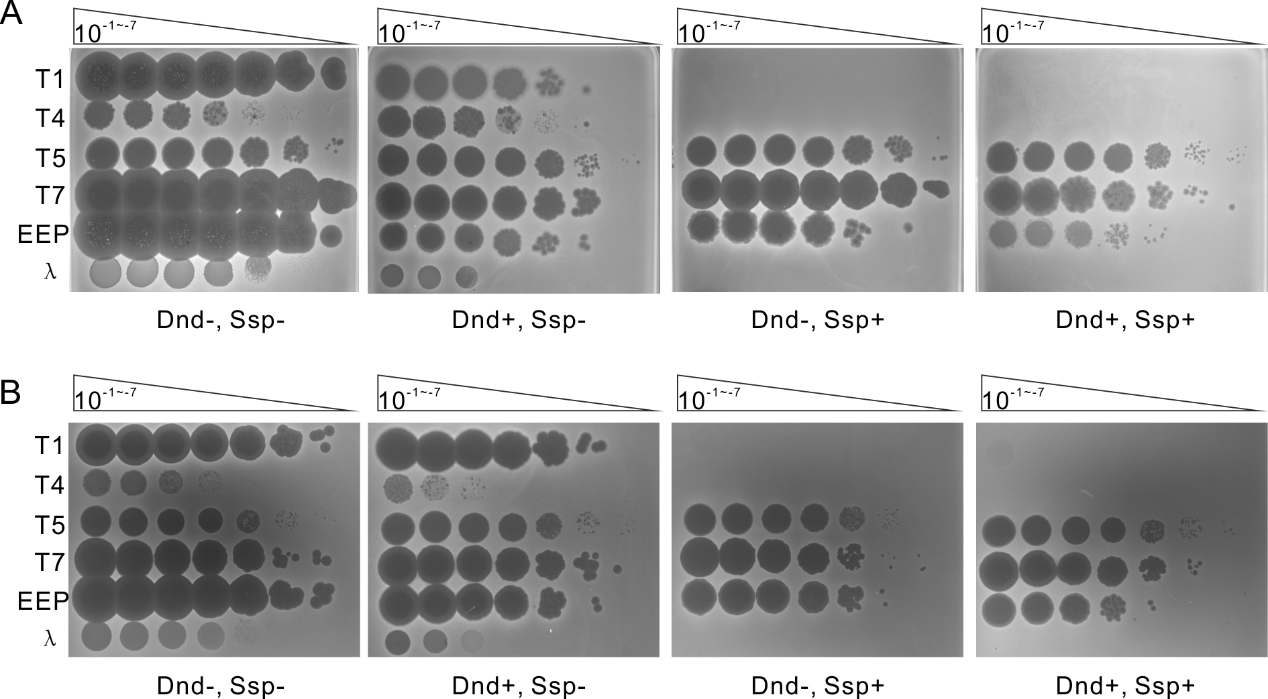
**
