# Supplementary material for: A DNA phosphorothioation-based Dnd defense system provides resistance against various phages and is compatible with the Ssp defense system
Source: mBio. 2023 Jun 1;14(4):e00933-23. doi: 10.1128/mbio.00933-23 (PMC10470545; doi:10.1128/mbio.00933-23)
Supplement: TABLE S1 — Strains and phages used in this study. [file mbio.00933-23-s0008.docx]

**TABLE S1. Strains and phages used in this study.**

| **Name** | **Characteristics** | **Source or reference** |
| --- | --- | --- |
| **Strains** |  |  |
| ***E. coli*** |  |  |
| DH10B | F–*mcrA*Δ(*mrr-hsdRMS-mcrBC*) φ80*lacZ*ΔM15 Δ*lacX74 recA1 endA1 araD139*Δ (*ara-leu*)7697 *galU galK*λ–*rpsL*(Str^R^) *nupG* | Invitrogen |
| BW25113 | *lacI*^+^*rrnB*_T14_ Δ*lacZ*_WJ16_ *hsdR*514 Δ*araBAD*_AH33_ Δ*rhaBAD*_LD78_ *rph-1* Δ*(araB–D)567* Δ*(rhaD–B)568* Δ*lacZ4787*(::*rrnB-3*) *hsdR514* *rph-1* | (1) |
| JW3350 | BW25113 derivative, *dam* deficient strain | Keio collection (2) |
| MG1655 | K-12 F^–^ λ^–^ *ilvG*^–^ *rfb-50* *rph-1* | (3) |
| MG1655-PT | MG1655 int(*aslA-glmZ*)::*sspBCDE* | (4) |
| B7A | *dndB-H*, d(G_PS_A)/d(G_PS_T) | (5) |
| 3234/A | *sspBCDE*, d(C_PS_C) | ATCC |
| ***S. enterica*** |  |  |
| *S. enterica* serovar Cerro 87 | Wild type, *dndB-H*, d(G_PS_A)/d(G_PS_T) | (5) |
| XTG103 | Cerro 87 derivative, *dndB-H* deletion mutant | (5) |
| ***P. mirabilis*** |  |  |
| *P. mirabilis* 1166 PMIR | *dndB-H*, d(G_PS_A)/d(G_PS_T) | (5) |
| ***B. marisrubri*** |  |  |
| *B. marisrubri* RED65 | *dndB-H*, d(G_PS_A) | (6) |
| **Phages** |  |  |
| T1 | *E. coli* phages, *Siphoviridae*, lytic | (7) |
| CC20 | T1 derivative, *dam* deficient strain | This work |
| T4 | *E. coli* phages, *Myoviridae*, lytic | (7) |
| T5 | *E. coli* phages, *Siphoviridae*, lytic | (7) |
| T7 | *E. coli* phages, *Podoviridae*, lytic | (7) |
| EEP | *E. coli* phages, *Siphoviridae*, lytic | (7) |
| λ | *E. coli* phages, *Siphoviridae*, lysogenic | (7) |
| λ*c*I857 | λ derivative, could be induced at 42 ℃ | New England Biolabs |
| PT1 | *S. enterica* phages, *Myoviridae*, lytic | (8) |

**References**

1. Grenier F, Matteau D, Baby V, Rodrigue S. 2014. Complete Genome Sequence of Escherichia coli BW25113. Genome Announc 2.

2. Baba T, Ara T, Hasegawa M, Takai Y, Okumura Y, Baba M, Datsenko KA, Tomita M, Wanner BL, Mori H. 2006. Construction of Escherichia coli K-12 in-frame, single-gene knockout mutants: the Keio collection. Mol Syst Biol 2:2006.0008.

3. Hayashi K, Morooka N, Yamamoto Y, Fujita K, Isono K, Choi S, Ohtsubo E, Baba T, Wanner BL, Mori H, Horiuchi T. 2006. Highly accurate genome sequences of Escherichia coli K-12 strains MG1655 and W3110. Mol Syst Biol 2:2006.0007.

4. Zou X, Xiao X, Mo Z, Ge Y, Jiang X, Huang R, Li M, Deng Z, Chen S, Wang L, Lee SY. 2022. Systematic strategies for developing phage resistant Escherichia coli strains. Nat Commun 13:4491.

5. Xu T, Yao F, Zhou X, Deng Z, You D. 2010. A novel host-specific restriction system associated with DNA backbone S-modification in Salmonella. Nucleic Acids Res 38:7133-7141.

6. Wang L, Chen S, Vergin KL, Giovannoni SJ, Chan SW, DeMott MS, Taghizadeh K, Cordero OX, Cutler M, Timberlake S, Alm EJ, Polz MF, Pinhassi J, Deng Z, Dedon PC. 2011. DNA phosphorothioation is widespread and quantized in bacterial genomes. Proc Natl Acad Sci U S A 108:2963-2968.

7. Xiong XL, Wu G, Wei Y, Liu LQ, Zhang YB, Su R, Jiang XY, Li MX, Gao HY, Tian XH, Zhang YZ, Hu L, Chen S, Tang Y, Jiang SS, Huang RL, Li ZQ, Wang YF, Deng ZX, Wang JW, Dedon PC, Chen S, Wang LR. 2020. SspABCD-SspE is a phosphorothioation-sensing bacterial defence system with broad anti-phage activities. Nature Microbiology 5:917-+.

8. Wu D, Tang Y, Chen S, He Y, Chang X, Zheng W, Deng Z, Li Z, Wang L, Wu G, Chen S. 2022. The functional coupling between restriction and DNA phosphorothioate modification systems underlying the DndFGH restriction complex. Nature Catalysis 5:1131-1144.
