# Supplementary material for: A DNA phosphorothioation-based Dnd defense system provides resistance against various phages and is compatible with the Ssp defense system
Source: mBio. 2023 Jun 1;14(4):e00933-23. doi: 10.1128/mbio.00933-23 (PMC10470545; doi:10.1128/mbio.00933-23)
Supplement: TABLE S2 — Plasmids and primers used in this study. [file mbio.00933-23-s0009.docx]

**TABLE S2. Plasmids and primers used in this study.**

| **Plasmids** | **Characteristics** | **Source or reference** |
| --- | --- | --- |
| pBluescript II SK(+) | Cloning vector, Amp^r^ | (1) |
| pWHU710 | pBluescript II SK(+) derivative carrying the homologous sequences of *dam* from T1 | This work |
| pACYC184 | Cloning vector, Cm^r^ | (2) |
| pWHU4386 | pACYC184 derivative carrying *dndB-H* from *P. mirabilis* 1166 PMIR | This work |
| pWHU4387 | pACYC184 derivative carrying *dndB-H* from *E. coli* B7A | This work |
| pWHU4388 | pACYC184 derivative carrying *dndB-H* from *Bermanella marisrubri* RED65 | This work |
| pWHU3638 | pACYC184 derivative carrying *sspBCDE* from *E. coli* 3234/A | This work |
| pCas9 | pACYC184 backbone carrying *cas9*, Cm^r^ | Addgene |
| pWHU711 | pCas9 derivative integrated spacer sequence targeting *dam* of T1 | This work |
| **Primers** | **Sequence (5’-3’)** | |
| **Construction of pWHU4386** | | |
| 1166-F | TTTGACAGCTTATCATCGATAAGCTTTTGTTCAGGTGTTTGTTGTTCC | |
| 1166-R | CACGATGCGTCCGGCGTAGAGGATCCTTTTCCCCACTTTTCAGTCTTC | |
| **Construction of pWHU4387** | | |
| B7A-LL | TTTGACAGCTTATCATCGATAAGCTTACCGATTTAGAGTTTTTCCAGTAGC | |
| B7A-LR | GGGAGTTAATGAAATGAAAATAAATACTACAGATCCAGATTTGAGG | |
| B7A-RL | CCTCAAATCTGGATCTGTAGTATTTATTTTCATTTCATTAACTCCC | |
| B7A-RR | CACGATGCGTCCGGCGTAGAGGATCCAAGTGATTAGCCCGTTTGATTTTGC | |
| **Construction of pWHU4388** | | |
| RED-LL | TTTGACAGCTTATCATCGATAAGCTTGTCGAGACTAGCAAGTGGGCTTG | |
| RED-LR | TGCTTGACTTTACCATGCTCATCAG | |
| RED-RL | CTGATGAGCATGGTAAAGTCAAGCA | |
| RED-RR | CACGATGCGTCCGGCGTAGAGGATCCTTTACTCCAGAAGCATTACAAAAT | |
| **Construction of pWHU3638** | | |
| 3234-F | CCACACCCGTCCTGTGGATCCTTTCCTCATACGAAGCTCTATTA | |
| 3234-R | CTCTCAAGGGCATCGGTCGACGGTATCGATAAGCTTCGTTACTG | |
| **Construction of pWHU710** | | |
| Dam-LL | GTCGACGGTATCGATAAGCTTATGAAAGACTTTAATGATAT | |
| Dam-LR | ATGCTTGTGATTGGGATGTATTCGCGAGTGAATGAGCAAC | |
| Dam-RL | GTTGCTCATTCACTCGCGAATACATCCCAATCACAAGCAT | |
| Dam-RR | CGCTCTAGAACTAGTGGATCCTCATACTTTCCTCACCTTTG | |
| **Construction of pWHU711** | | |
| Spacer-F | AAACCAAGAAGGCCATTGAGCAAAG | |
| Spacer-R | AAAACTTTGCTCAATGGCCTTCTTG | |
| **Verification of λ lysogenization** | | |
| Lambda-F | GCAATGCGGCGTTATAAGCA | |
| Lambda-R | TCCTGTTGATAGATCCAGTA | |
| **qPCR for T7** | | |
| RT-T7-F | CCGAGATGAGGTAGGTGGTC | |
| RT-T7-R | CTGCGAGTAACACCGTAAGC | |
| RT-E-gapA-F | TTTCCGTGCTGCTCAGAAAC | |
| RT-E-gapA-R | GTCAACACCAACTTCGTCCC | |
| **qPCR for PT1** | | |
| RT-PT1-F | ATGGGCCTGTTCATCTGTGA | |
| RT-PT1-R | AAACTTCGTGCCTTTGGTCC | |
| RT-S-gapA-F | TAACCTGAAATGGGACGAAG | |
| RT-S-gapA-R | GGCCTTCGTATTTGTCAAAG | |

**References**

1. Alting-Mees MA, Short JM. 1989. pBluescript II: gene mapping vectors. Nucleic Acids Res 17:9494.

2. Rose RE. 1988. The nucleotide sequence of pACYC184. Nucleic Acids Res 16:355.
